# SVHunter: long-read-based structural variation detection through the transformer model

**DOI:** 10.1093/bib/bbaf203

**Published:** 2025-05-09

**Authors:** Runtian Gao, Heng Hu, Zhongjun Jiang, Shuqi Cao, Guohua Wang, Yuming Zhao, Tao Jiang

**Affiliations:** College of Life Science, Northeast Forestry University, Harbin 150000, China; College of Computer and Control Engineering, Northeast Forestry University, Harbin 150000, China; College of Life Science, Northeast Forestry University, Harbin 150000, China; College of Computer and Control Engineering, Northeast Forestry University, Harbin 150000, China; College of Life Science, Northeast Forestry University, Harbin 150000, China; College of Computer and Control Engineering, Northeast Forestry University, Harbin 150000, China; School of Computer Science and Technology, Harbin Institute of Technology, Harbin 150001, China; College of Computer and Control Engineering, Northeast Forestry University, Harbin 150000, China; School of Computer Science and Technology, Harbin Institute of Technology, Harbin 150001, China; College of Computer and Control Engineering, Northeast Forestry University, Harbin 150000, China; School of Computer Science and Technology, Harbin Institute of Technology, Harbin 150001, China

**Keywords:** structural variation, long-read sequencing, transformer model, dynamical clustering

## Abstract

Structural variations (SVs) are genomic rearrangements larger than 50 bp, that are widely present in the human genome and are associated with various complex diseases. Existing long-read-based SV detection tools often rely on fixed rules or heuristic algorithms, which can oversimplify the complexity of SV signatures. Therefore, these methods usually lack flexibility and cannot fully capture SV signals, leading to reduced accuracy and robustness. To address these issues, we propose SVHunter, a transformer-based method for long-read SV detection. SVHunter combines convolutional neural networks and transformers to capture both local and global SV signatures, enabling accurate identification of SVs. Additionally, SVHunter employs the mean shift clustering algorithm, which dynamically adjusts bandwidth parameters to accommodate different types of SVs without requiring a preset number of clusters, thus allowing precise breakpoint clustering. Validation across multiple sequencing platforms and datasets demonstrates that SVHunter excels at detecting various types of SVs, with a notable reduction in the false discovery rate. This highlights considerable strong potential for both research and clinical applications.

## Introduction

Structural variations (SVs) are genomic rearrangements longer than 50 bp, with common types including deletion (DEL), insertion (INS), inversion (INV), duplication (DUP), and translocation (TRA) [1]. As one of the major sources of variation in the human genome, SVs affect a far greater number of base pairs than single nucleotide variants and short insertions/deletions [2–4], contributing more significantly to individual genomic differences. SVs not only are a key source of genomic diversity but are also closely associated with various major diseases, such as autism [5], schizophrenia [6], and Alzheimer’s disease [7]. Therefore, accurately detecting these variations is crucial for understanding their roles in disease. The development of more efficient SV detection methods is highly importance for uncovering their biological significance and clinical relevance.

The mainstream approaches include next-Generation sequencing (NGS) [8] and third-generation sequencing (TGS) [9]. NGS, with a sequencing accuracy of up to 99% and relatively low cost, can generate short read sequences ranging from 150 to 500 bp, which are suitable for large-scale detection of genomic variations. In recent years, various NGS-based SV detection methods have been developed, with common algorithms such as Manta [10], GRIDSS [11], and SV-channels [12]. These tools infer SVs via paired-end sequences, read depth variations, and split reads spanning breakpoints. According to previous studies [12], Manta, e.g., is notable for its high recall, particularly for deletions, while GRIDSS achieves high precision across various SV types by integrating multiple evidence types, including assembly-based approaches. In addition to traditional algorithm-based methods, deep learning approaches for short-read sequencing have also been developed. Sv-channels utilizes a one-dimensional convolutional neural network (CNN) to enhance the precision of short-read SV detection by transforming sequencing data into simplified 1D ‘channels’ representations. These channels are feature arrays centered on candidate SV breakpoints, specifically capturing localized signals. In contrast, our study employs convolutional filters that operate on fixed-size genomic windows, independent of predefined breakpoints, to learn broader sequence patterns. However, previous studies [13–16] have shown that approximately half of germline SVs are associated with repetitive sequences, whereas short-read methods have a recall rate of only 30%–75%. Hence, these NGS-based approaches remain insufficient for detecting large SVs in complex genomic regions and repetitive sequences.

TGS largely compensates for the limitations of NGS. TGS technologies, such as those of Pacific Biosciences (PacBio) [17] and Oxford Nanopore Technologies (ONT) [18], can generate long-read sequences spanning thousands to millions of base pairs. Compared with NGS, the long-read capability of TGS allows it to span entire SV regions, providing significant advantages in aligning repetitive genomic regions, identifying complex SVs, and detecting large SVs. However, TGS is not without its challenges. Its relatively high sequencing error rate (typically between 5% and 20%) can increase the risk of false-positive, particularly in the alignment of complex genomic regions [19–21]. To address these challenges, several alignment-based heuristic algorithms have been developed [22], such as cuteSV [23], Sniffles2 [24], and SVIM [25]. These algorithms infer SVs by analyzing the alignment information of long reads. However, because they rely on manually designed heuristic rules, they can be prone to noise in certain situations. For instance, sequencing errors or misalignments may be mistaken for true SV signals. In the case of complex SV events, noise may obscure the actual signal distribution, leading to inaccurate breakpoint localization or biased variant length estimates.

In recent years, with the rapid development of deep learning technologies, their application in SV detection has begun to emerge. Unlike traditional heuristic algorithms, deep learning methods can automatically learn complex features from large-scale labeled data, thereby better capturing the latent patterns of SVs. Several studies have demonstrated the potential of deep learning in SV detection [26]. For example, SVision detects complex SVs by extracting alignment features and generating images [27], MAMnet focuses on detecting two types of SVs—deletions and insertions—through feature extraction [28]. Deep learning methods have shown great promise in SV detection, particularly in handling complex variations and noise filtering. By automatically learning features, deep learning can capture more subtle variant signals, significantly improving detection accuracy and flexibility. However, current deep learning-based SV calling methods still face challenges; for instance, the image generation step may result in the loss of important sequence information, and a relatively limited range of SV types can be identified. As a result, the power of deep learning has not yet been fully unleashed in SV calling tasks.

To address the limitations of existing SV detection methods, we developed SVHunter, a transformer-based approach for detecting SVs from long reads. Unlike traditional heuristic algorithms, SVHunter combines local feature encoding with global dependency modeling, enabling it to capture more complex variant patterns. Specifically, it uses a CNN [29] to encode local variant features, effectively capturing multiscale dependencies and providing high-quality inputs for global modeling. Simultaneously, it leverages the transformer [30] network to model global dependencies between submatrices, further improving its SV recognition capabilities. SVHunter supports the detection of all five major SV types—DEL, INS, inversions (INV), duplications (DUP), and translocations (TRA)—and uses mean shift [31] clustering to precisely localize breakpoints, increasing both detection accuracy and sensitivity. The combination of these techniques allows SVHunter to improve detection precision and reduce the number of false-positive, delivering more reliable results for genomic research and clinical applications. By leveraging the strengths of the transformer architecture, SVHunter represents a significant advancement in SV detection and is poised to play an important role in future genomic studies and clinical diagnostics.

## Materials and methods

**Figure 1:**
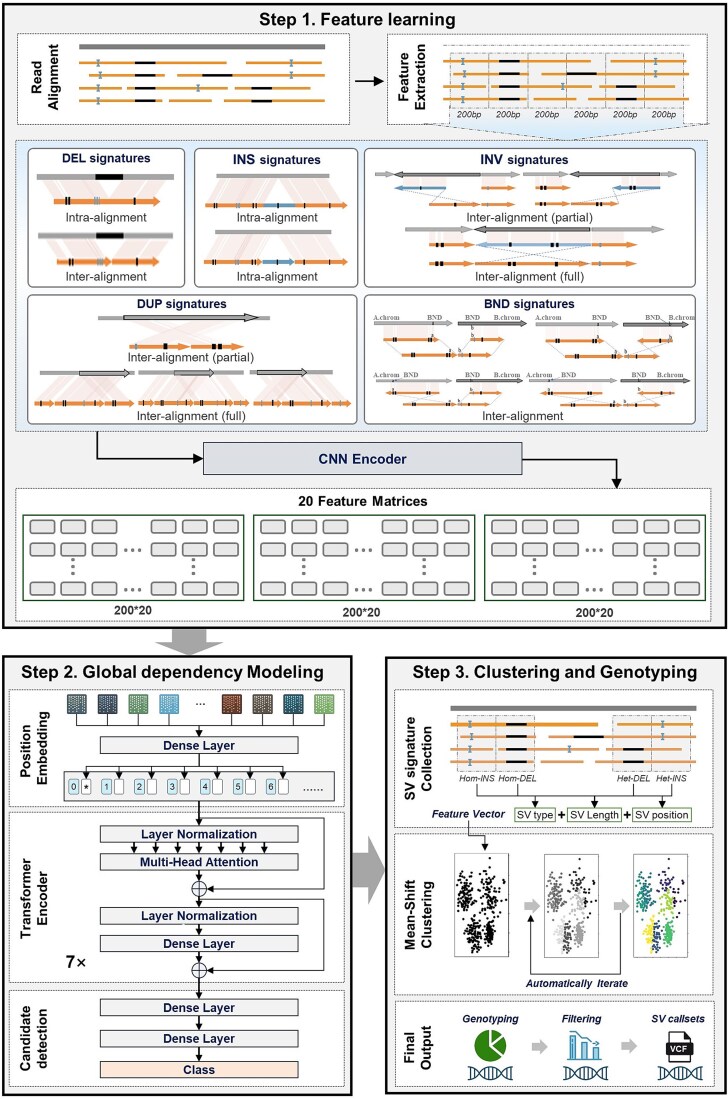
The overall workflow of SVHunter. SVHunter follows a three-step process for SV detection. In Step 1: Feature learning, SV-related features are extracted from read alignments and categorized into five major SV signatures: Deletions (DEL), insertions (INS), INV, DUP, and breakends (BND). These signatures are transformed into feature matrices using a CNN. In Step 2: Global dependency Modeling, a transformer-based model with position encoding and multi-head attention captures dependencies between SV features, refining candidate SV detection. Finally, in Step 3: Clustering and genotyping, SV signatures are clustered using a mean-shift algorithm, followed by genotyping and filtering to produce a high-confidence variant call set in VCF format.

SVHunter is a transformer-based method for accurate SV detection, significantly enhancing both detection accuracy and robustness. The workflow is illustrated in Fig. 1. First, SVHunter extracts variant features from the alignment of long-read sequencing data to a reference genome. The data are divided into 200 bp submatrices, where local feature encoding is performed using via CNN to capture variation patterns at the local scale. Next, multiple submatrices are concatenated into a 2000 bp feature matrix, and a transformer network is applied to model global dependencies across submatrices, enabling the precise identification of variant regions over longer sequences. Once potential variants are detected, SVHunter employs the mean shift clustering algorithm to refine breakpoint positions. This unsupervised method analyzes variant sites, ensuring high-precision localization of breakpoints. Finally, SVHunter determines the genotype of each variant through a voting mechanism that integrates three genotype determination strategies. After an automated read support selection and filtering step, SVHunter generates standardized VCF files, ensuring both accuracy and consistency in the final detection results.

### Feature learning

The feature learning module of SVHunter captures deep variant information by extracting and deeply encoding variant features from BAM files. Feature learning consists of two main steps: feature extraction and feature encoding.

#### Feature extraction

During the feature extraction phase, SVHunter extracts 10 key variant features at each site from the BAM file, accounting for both forwards and reverse alignment information, resulting in a total of 20 features. Detailed descriptions of these features are provided in the supplementary notes. For each site, whenever a specific variant feature is detected, its corresponding count is incremented. The forwards and reverse alignment features are then combined, producing the 20 features used as input for the subsequent deep learning model.

#### Feature encoding

After feature extraction, SVHunter employs CNNs combined with a channel and spatial attention mechanism, CABM [32] to encode the extracted features (Supplementary Fig. S3). CNNs effectively capture local variant features through convolution operations, and are particularly adept at handling variant signals with spatial dependencies. The convolution operations identify variant features in local regions, and by sharing convolutional kernel weights, they enhance parameter efficiency and reduce training complexity [33].

In practice, SVHunter divides the feature matrix into 200 bp submatrices, which are processed through CNNs and the attention mechanism. Each submatrix is encoded using these mechanisms, generating feature representations that are subsequently utilized for global dependency modeling and variant detection.

### Global dependency modeling

After completing local feature encoding, SVHunter proceeds to identify SV regions through global dependency modeling. In this step, multiple local features are aggregated into larger feature blocks to capture variant signals spanning longer sequences. Specifically, SVHunter combines 10 submatrices of 200 bp into a single 2000 bp feature matrix, enabling the observation of sequence features over a larger window. This facilitates the detection of variant patterns that extend across multiple submatrices.

SVHunter employs a transformer network to model the global dependencies between feature blocks, identifying the relationships among them. The key strength of the Transformer lies in its ability to capture long-range dependencies through its self-attention mechanism. Unlike traditional CNNs or RNNs, the transformer can dynamically attend to any part of the sequence, making it particularly well-suited for handling SV signals that span large genomic regions [34, 35].

In practice, SVHunter first applies positional encoding to each 200 bp submatrix, allowing the network to recognize their relative positions within the feature blocks. A multihead self-attention mechanism is then used to capture dependencies between different submatrices. After processing through the transformer network, SVHunter accurately predicts potential variant regions in the genome. The global features of each submatrix are classified through a fully connected layer, and the model determines the presence of SVs on the basis of the predicted probabilities. If multiple adjacent regions are predicted to contain variants, SVHunter automatically merges them into a larger variant region. The merging process can be formalized as follows:


(1) \begin{equation*} Merge\left(R1,R2\right)=\left[\mathit{\min}\left({R}_1^{start},{R}_2^{start}\right),\mathit{\max}\left({R}_1^{end},{R}_2^{end}\right)\right] \end{equation*}

where *R_1_* and *R_2_* are the start and end points, respectively, of two adjacent variant regions. Through this approach, SVHunter effectively combines local and global features to detect SVs spanning multiple submatrices, thereby improving both the comprehensiveness and accuracy of detection.

### Precise breakpoint clustering

The detection of SVs often relies on clustering features from the same SV event to enhance the signal and reduce noise. However, traditional clustering methods frequently face challenges. These methods typically based on fixed distance metrics, making it difficult to flexibly handle the varying strengths and distributions of variant signals, especially across different types of SVs. To overcome these limitations, SVHunter employs a mean shift clustering algorithm based on kernel density estimation (KDE), which adapts dynamically to the data distribution. Unlike fixed-metric approaches, SVHunter adjusts the bandwidth parameters on the basis of the average error rate of the reads and the SV type, allowing it to better capture variant signals across a range of genomic contexts.

Due to memory and computational constraints, SVHunter does not retain full long-read sequences throughout the deep learning inference step. Instead, it extracts relevant variant features and defers read-level reprocessing until after candidate SV regions have been identified. Once precise variant regions are identified, SVHunter analyzes CIGAR strings and split-read information from alignment files to extract SV types within the region, and then applies mean shift clustering, a density-based clustering algorithm that groups variants based on their positional proximity. In mean shift clustering, the bandwidth parameter defines the search radius around each data point, determining how closely variants must be located to be grouped into the same cluster. A larger bandwidth results in fewer, broader clusters, while a smaller bandwidth leads to more, finer-grained clusters. SVHunter dynamically adjusts the bandwidth parameters based on the average error rate of the reads and the SV type. For example, a bandwidth of 1000 is set for DEL, 300 for INS, and 500 for DUP and INV. For high-quality reads with lower error rates, the bandwidth is increased to 1500 to capture variant signals over a broader range. Since TRA involve two breakpoints, they are clustered directly based on positional information rather than using bandwidth-based density estimation.

After clustering, SVHunter further refines the results by calculating the median length of the variants within each cluster and using this as a reference to subdivide the variants into more granular subclusters. Specifically, all the variants are sorted by length, and the median is calculated. A preset threshold ratio (default: 0.7) is then applied to the median to determine whether two variants should be placed in the same subcluster. If the difference in length between two variants is smaller than the median multiplied by the threshold ratio, they are grouped into the same subcluster. Otherwise, they are separated into new subclusters.

In the final step, SVHunter ensures that the variants within the same cluster exhibit similar length characteristics through this refined clustering method, thus enhancing both the accuracy and robustness of the clustering process.

### Automatic selection of supporting reads

For different types of SVs, SVHunter employs a dynamic formula to evaluate the supporting reads within each variant cluster and determine the presence of SVs. This formula comprehensively considers both global and local coverage, error rates, and other relevant information, dynamically adjusting the minimum number of supporting reads for each type of SV. This ensures that different types of SVs can be accurately detected.


(2) \begin{equation*} mi{n}_{support}=\mu \ast{C}_{global}^{\varphi}\ast \left(1+\rho \ast \mathit{\tanh}\left(\frac{C_{local}-{C}_{global}}{C_{global}}\right)\right) \end{equation*}

The global average coverage (*C_global_*) refers to the average sequencing depth across the entire genome, whereas the local coverage (*C_local_*) represents the sequencing depth in the specific region where the variant is located. The formula also incorporates a global adjustment factor (*μ*) to modulate the influence of global coverage, and a coverage power exponent (*σ*) to control the weight of coverage. Additionally, a local coverage deviation factor (*ρ*) accounts for the deviation between local coverage and global coverage allowing SVHunter to adjust for regional variations in sequencing depth. The read error rate is also factored into the formula, ensuring that fewer supporting reads are required when the error rates are low.

This formula dynamically adjusts the minimum number of supporting reads by comparing the difference between local coverage and global coverage. In practice, SVHunter extracts coverage information from the BAM file for the variant region and dynamically adjusts the minimum number of supporting reads based on the deviation between local coverage and global coverage. For reads with an error rate lower than 0.1, SVHunter uses the standard formula to calculate the number of supporting reads. In cases of high error rates or low global coverage, the formula adjusts the parameters accordingly to ensure robust detection.

### Genotyping by voting mechanism

Genotyping refers to the process of determining the specific genetic variant (or allele combination) present at a given locus in a sample. In the context of structural variant (SV) detection, SV genotype represents the allelic state of an SV in a diploid genome, typically classified into three categories: homozygous reference (0/0), heterozygous (0/1), and homozygous alternative (1/1). SVHunter employs multiple methods to accurately infer the genotype of a sample, integrating likelihood-based probabilistic inference, Bayesian inference [36], the expectation–maximization (EM) algorithm [37], and a read support ratio method. The final genotype is selected through a voting mechanism that combines the results from these methods, enhancing both robustness and accuracy.

SVHunter first evaluates the likelihood of each genotype (i.e., 0/0, 0/1, 1/1) by calculating the likelihood values for different genotypes. It assumes that the supporting reads for the reference allele and the variant allele follow a certain probability distribution, which is adjusted based on the sequencing error rate. Subsequently, SVHunter applies Bayesian inference, introducing prior probabilities to further optimize genotype determination. The genotype with the highest posterior probability is then selected as the preliminary result.

In parallel, SVHunter employs the EM algorithm to optimize genotype estimation. The EM algorithm iteratively refines the probability distribution of genotypes. During the expectation step, SVHunter calculates the expected values based on the current parameter estimates, while in the maximization step, the probability distribution of the genotypes is updated. After several iterations, the algorithm converges to the most likely estimates for the 0/0, 0/1, and 1/1 genotypes.

Additionally, SVHunter utilizes a simplified method based on the read support ratio for genotype inference. If the proportion of supporting reads for the reference allele exceeds a set threshold (default: 0.7), the genotype is determined to be 0/0; if the variant allele has a higher proportion, the genotype is inferred as 1/1. If the ratio is ~ 50%, the genotype is determined as 0/1.

Finally, to enhance robustness, SVHunter integrates these multiple methods via a voting mechanism. The results from Bayesian inference, the EM algorithm, and the read support ratio method are combined, and the genotype with the most votes is selected as the final result. This ensemble approach reduces potential errors from any single method, improving the accuracy of genotype detection.

## Results

### Benchmark results on real sample

The ability to detect SVs in real samples is crucial. To achieve this, we first selected the well-studied human sample HG002, published by the Genome in a Bottle (GIAB) project [38], to evaluate SVHunter performance across different sequencing platforms and coverage levels. Specifically, we used data from three sequencing platforms—PacBio CLR, CCS, and ONT—to systematically compare the performance of SVHunter with that of mainstream tools, including Sniffles2, SVIM, and cuteSV. These tools were chosen because they are among the most widely used and well-established SV callers for long-read sequencing. They have been extensively benchmarked across multiple datasets, exhibit robust performance across different sequencing technologies, and are actively maintained with continuous improvements.

**Figure 2:**
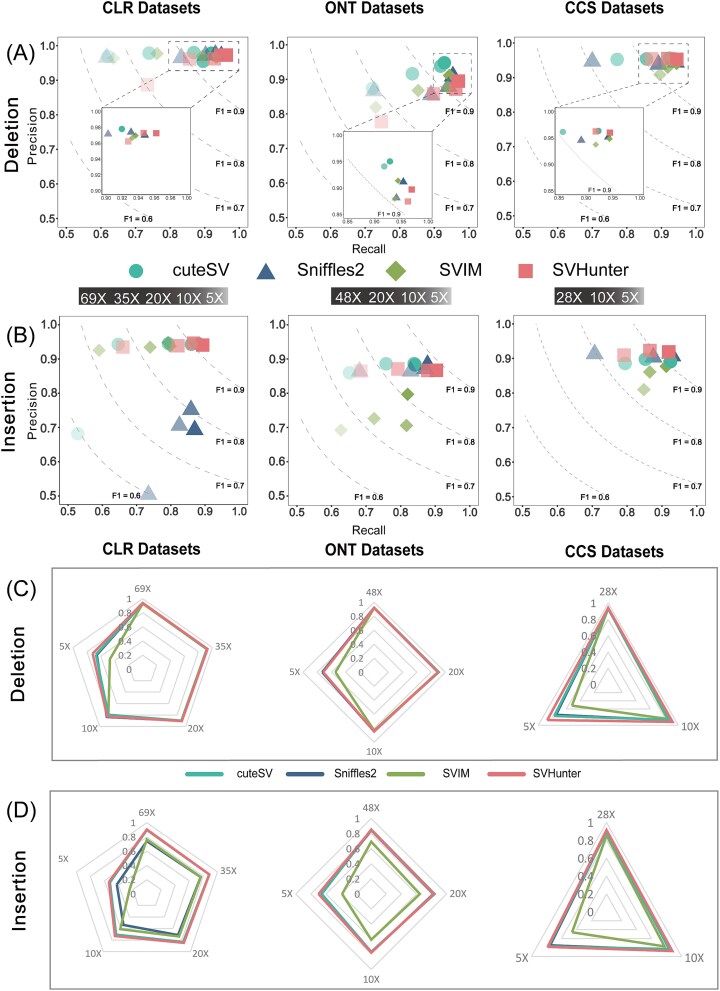
Detection results on the GIAB HG002 SV dataset, comparing performance across different sequencing platforms and coverage. (A) DEL results at different coverages. (B) INS results at different coverages. (C) DEL genotype results at different coverages. (D) INS genotype results at different coverages.

Under the original high-coverage conditions (i.e., PacBio CLR: 69×, CCS: 28×, and ONT: 48×), SVHunter was superior in detecting DEL and INS, as shown in Figure 2. For the 69× PacBio CLR data, SVHunter achieved F1 scores of 91.62% for INS and96.75% for DEL, outperforming Sniffles2 (INS: 77.13%, DEL: 95.90%), SVIM (INS: 86.30%, DEL: 95.30%), and cuteSV (INS: 91.13%, DEL: 94.80%). Similarly, SVHunter continued to perform well on the 48× ONT and 28× CCS datasets, with the F1 scores of ONT being 88.42% for INS and 93.20% for DEL, and those of CCS being 91.91% for INS and 95.23% for DEL. Owing to the strong representational capacity of its transformer model and the robustness of the mean shift clustering algorithm, SVHunter maintained stable performance even on downsampled, lower-coverage datasets. Notably, at 5× coverage, SVHunter’s results still surpassed those of the other tools. In addition to long-read sequencing, we also compared the structural variant detection results from short-read sequencing tools, including Manta, Gridss, and SV-channels, on the same HG002 sample. The results of these tools are provided in Supplementary Table S3 for reference. Furthermore, to evaluate SV detection performance across different species, we conducted an additional benchmark using real long-read data from *Arabidopsis thaliana*. The detailed methodology and results are provided in Supplementary Table S18. Owing to its higher overall precision, SVHunter achieved an F1 score that exceeded that of the second-best-performing tool, Sniffles2, by 4.76%, thereby highlighting its robustness and effectiveness in structural variant detection across different species. In genotype validation experiments, SVHunter demonstrated strong adaptability across multiple platforms and coverage depths, consistently maintaining high precision and recall, further validating its reliability in variant detection.

In brief, in benchmark testing on the HG002 sample, SVHunter demonstrated outstanding variant detection capabilities across both high-quality PacBio CCS data and high-error-rate ONT and CLR data. This performance is largely attributed to its transformer model’s efficiency in handling complex signals and the robustness of the mean shift clustering algorithm in variant clustering. These results show that SVHunter consistently maintains high precision, recall, and F1 scores across different sequencing platforms and coverage depths, validating its practicality and reliability in real-world datasets.

### Benchmark results on simulated sample

**Figure 3:**
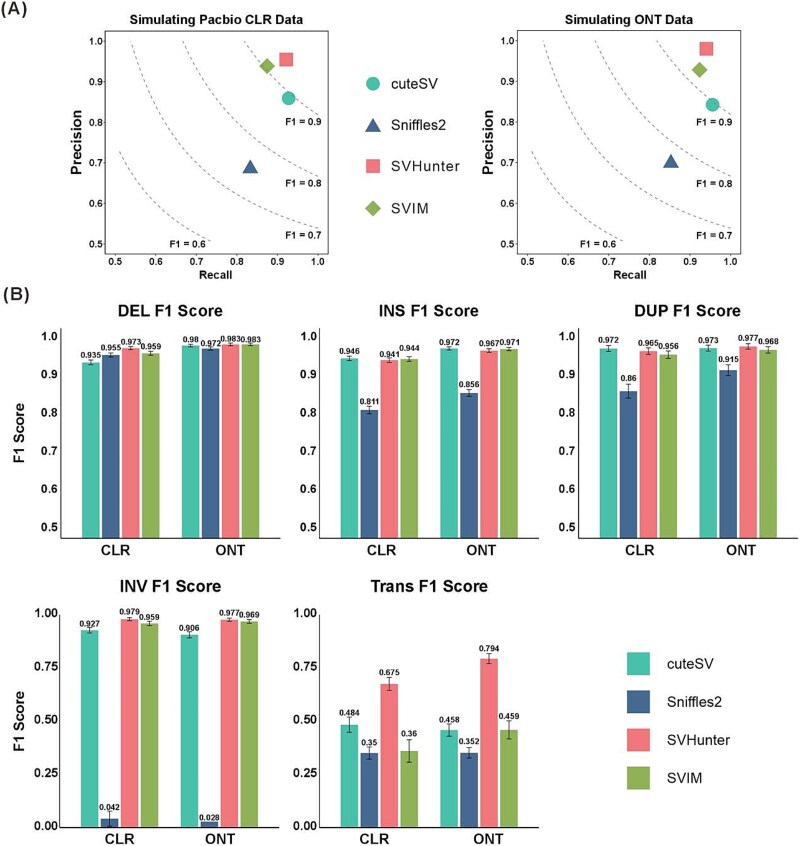
Results on the simulated dataset. (A) Precision and recall for PacBio CLR and ONT data. (B) F1 scores for different variant types (DEL, INS, DUP, INV, and TRA) across CLR and ONT simulated datasets, comparing the performance of different tools.

We have previously evaluated SVHunter’s performance on the real HG002 benchmark dataset, demonstrating its robustness across multiple sequencing platforms and different coverage depths. To further assess SVHunter’s generalizability and performance across different variant types, we utilized a simulated dataset comprising various SV types (including INS, DEL, DUP, INV, and TRA). Simulated datasets provide precise ground truth information, allowing a more comprehensive assessment of each tool’s accuracy and recall across a broader range of variant types. We used SURVIVOR [39] and PBSIM2 [40] to simulate both CLR and ONT datasets and compared the results with those of Sniffles2, SVIM, and cuteSV. As shown in Fig. 3, SVHunter achieved the highest F1 scores of 93.78% and 94.35% on the CLR and ONT datasets, respectively—improvements of 3.25% and 1.19% over the second-best tool, SVIM. This demonstrates SVHunter’s superiority in detecting a wide range of SVs. Additionally, to evaluate SV detection performance across different species, we simulated long-read data for *A. thaliana*. The specific methods and results are detailed in Supplementary Tables S16 and S17. Similar to the results obtained from real data, SVHunter consistently achieved the highest F1 score across multiple simulation replicates, which was primarily attributed to its higher precision. The improvements ranged from 0.3% to 1.91% compared to other tools.

On the PacBio CLR and ONT datasets, SVHunter excelled in SV detection, particularly for DEL and INV. It achieved F1 scores of 97.29% (CLR) and 98.27% (ONT) for DEL. In INV detection, SVHunter demonstrated a clear advantage, with F1 scores of 97.91% (CLR) and 97.71% (ONT), significantly outperforming SVIM (CLR: 95.94%, ONT: 96.88% and cuteSV (CLR: 92.70%, ONT: 90.63%). Although SVHunter did not achieve the highest F1 scores for INS, it maintained a strong balance between precision and recall, and in the challenging TRA detection, it achieved significantly higher F1 scores than other tools, demonstrating robustness in complex variant scenarios. To address potential bias caused by TRA detection, we also calculated the weighted F1 scores across all SV types on ONT data. As shown in Supplementary Table S6, SVHunter still achieved the highest weighted F1 score, further validating its overall performance. Additionally, to analyze the impact of different SV distributions on the results, we conducted further experiments using simulated datasets with an equal number of SVs across variant types. SVHunter maintained its leading performance, achieving the highest F1 scores among all tools. Details of this experiment can be found in Supplementary Table S8.

Altogether, SVHunter’s performance on the simulated dataset further validated its superiority in detecting various types of SVs. For the five types of SV, SVHunter consistently demonstrated high precision and recall. In particular, it outperformed other mainstream tools for complex variants such as INV and TRA.

### Evaluation of false discovery rates on CHM13 sample

The performance on simulated datasets indicates that SVHunter has strong detection capabilities across various SV types. However, simulated data may not fully reflect a tool’s performance in handling complex real-world samples, particularly in terms of its ability to combat false-positive. With the maturation of telomere-to-telomere (T2T) assembly technologies, high-coverage de novo assemblies have been shown to detect more SVs with greater accuracy than traditional alignment methods. [41–43] To comprehensively assess SVHunter’s SV detection capabilities across different reference genomes, we used CHM13 (*Complete Hydatidiform Mole 13*) sample PacBio CLR 36× data and conducted two sets of alignment experiments: (i) aligning the data to the GRCh38 reference genome, and (ii) aligning the data to the CHM13 reference genome itself. As part of this evaluation, we also downsampled both datasets to 20× and 10× coverage to assess the impact of sequencing depth on SV detection performance (Supplementary Table S11 and Table S12). Through these two experiments, we evaluated the performance of four tools in detecting SVs in the commonly used reference genome (GRCh38) and their ability to mitigate false positives in the high-quality haploid genome (CHM13). The supplementary notes provide additional details on multiple aspects of our benchmarking methodology, including a discussion of the relative merits and drawbacks of assembly versus non-assembly-based method, the justification for using CHM13 as a gold-standard reference, and other relevant analyses.

**Figure 4:**
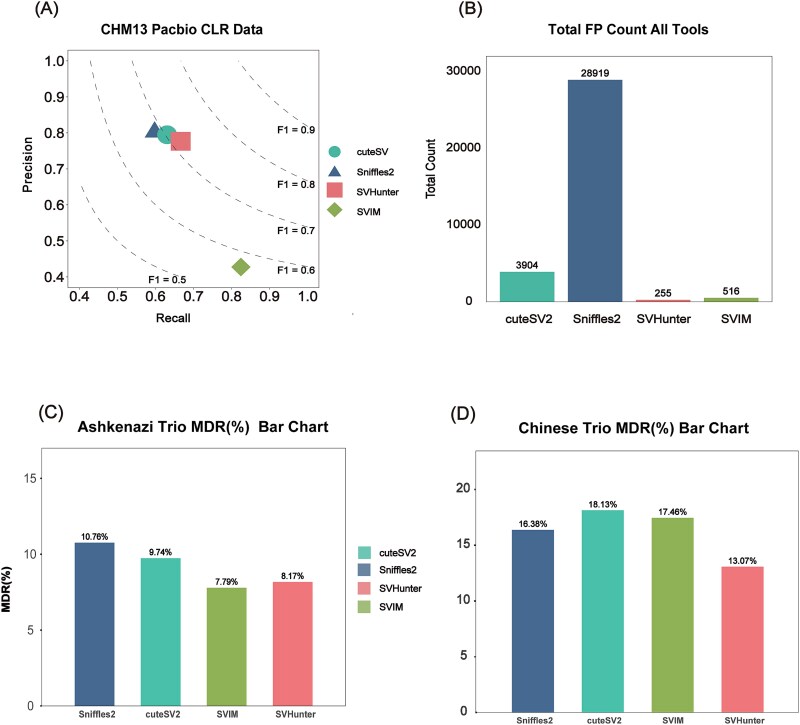
Results from the CHM13 and trio datasets. (A) Precision and recall for CHM13 PacBio CLR data aligned to GRCh38. (B) The number of false positives detected by comparing CHM13 to the CHM13 reference genome. (C) Mendelian discordance rate for the Ashkenazi trio. (D) Mendelian discordance rate for the Chinese trio.

In the first experiment, the CHM13 sample PacBio CLR 36× data were aligned to the GRCh38 reference genome, and the SV set generated by the de novo assembly-based Dipcall [44] tool was used as the benchmark set for comparison. Figure 4A shows the performance of each tool in terms of precision, recall, and F1 score. SVHunter demonstrated a well-balanced performance on this dataset, achieving the highest F1 score of 0.7172. This finding indicates that SVHunter was able to captured a large portion of the SVs while maintaining relatively high precision.

In the second experiment, the CHM13 PacBio CLR 36× data were aligned to the CHM13 reference genome to test false-positive resistance. Since CHM13 is a non-chimeric, error-free genome, an ideal tool should detect no variants. Figure 4B shows Sniffles2 detected 28,919 variants, which suggests a high number of false positives. Similarly, cuteSV and SVIM identified 3904 and 516 variants, respectively, indicating the potential for false-positive detections. By comparison, SVHunter detected only 255 variants, highlighting its superior resistance to false positives. This demonstrates that, compared to other algorithms, SVHunter is highly effective at filtering out false-positive SVs in highly repetitive regions, highlighting its strong false-positive resistance capabilities, likely due to its deep learning-based approach.

### Mendelian discordance rate test

Mendelian inheritance laws are an important theoretical basis for assessing the consistency of genetic variation and are widely applied in evaluating the performance of SV detection tools [45]. By calculating the mendelian discordance rate (MDR), we can measure the consistency of a tool when detecting variants in parent-offspring samples. To evaluate the performance of different SV detection tools, we used PacBio CLR sequencing data from two trios—Ashkenazi and Chinese—and developed scripts to calculate the MDR for each tool across various SV types. The performance of SVHunter, SVIM, Sniffles2, and cuteSV was assessed.

We performed SV detection on the data, and then calculated the MDR between the offspring and parental samples for each tool to evaluate their detection consistency. Figure 4C shows the MDR results for the Ashkenazi family, where SVHunter achieved an MDR of 8.17%, demonstrating excellent performance, second only to SVIM, and outperforming cuteSV and Sniffles2. Although SVIM achieved the lowest MDR, this was due to its lack of genotype information for TRA variants. Figure 4D shows the MDR results for the Chinese family. SVHunter achieved an MDR of 13.07%, which was lower than the results of the other three tools. The MDRs for Sniffles2, SVIM, and cuteSV were 16.38%, 17.46%, and 18.13%, respectively, indicating that SVHunter exhibited greater consistency and better resistance to false-positive in this family.

Overall, the MDR analysis of the two different trios demonstrated that SVHunter exhibited low MDRs in both the Ashkenazi and Chinese families. In contrast, SVHunter not only exhibited excellent performance in terms of MDR but also proved applicable across all variant types, showcasing its robustness and resistance to false-positive. Combining the results from both families, SVHunter demonstrated superior performance across diverse genomic backgrounds, confirming its broad applicability as an SV detection tool.

### Ablation experiments

SVHunter first encodes the data via a CNN and then captures global dependencies through a transformer for variant detection. To further validate the contribution of each module in SVHunter, we conducted ablation experiments, separately evaluating the CNN model, the transformer model, and the complete model combining both (ALL). To ensure a fair comparison, we analyzed model performance under both a fixed 20-epoch training schedule and an early stopping strategy. The first 20 epochs were used to provide a consistent comparison across all models, preventing discrepancies due to varying stopping points, while early stopping allowed each model to reach its optimal performance. The complete model reached early stopping at 53 epochs, the CNN ablation model at 27 epochs, and the Transformer ablation model at 52 epochs. In the main manuscript, figures only display results from the first 20 epochs to maintain clarity and facilitate direct visual comparisons. However, all results, including full training curves and final performance metrics under early stopping, are provided in the supplementary materials for a comprehensive analysis.

**Figure 5:**
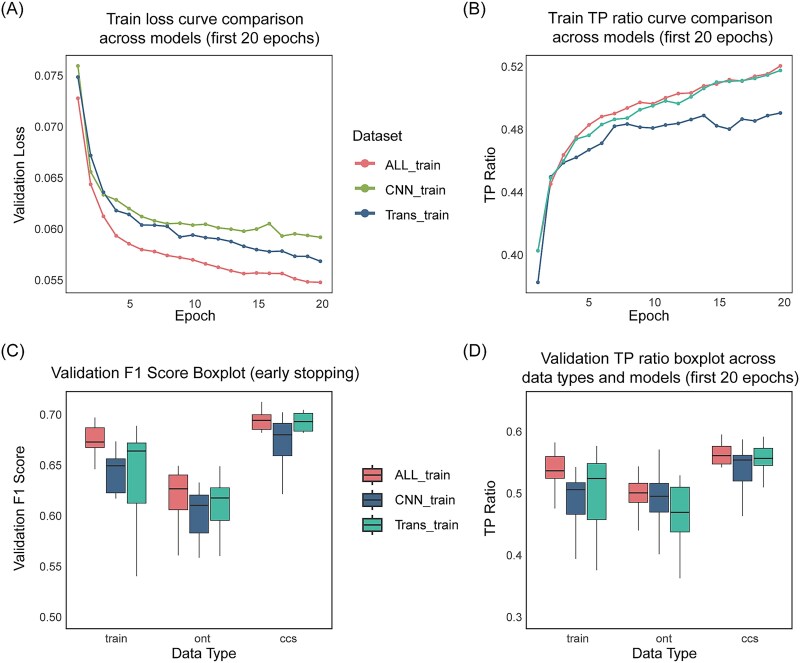
Ablation study results comparing the performance of different model configurations across sequencing platforms. (A) Training loss curves across different models. The results shown are based on the training datasets from three sequencing platforms: CLR, ONT, and CCS. (B) Training true positive (TP) ratio count curves. The results shown are based on the training datasets from three sequencing platforms: CLR, ONT, and CCS. (C) Distribution of validation F1 score. (D) Distribution of validation true positive ratio.

Based on the training loss curves (Fig. 5A), both the ALL and transformer models show smooth and rapid convergence, achieving lower loss values by the end of training. In contrast, the CNN model converges slower, with higher loss values persisting throughout, suggesting weaker representational capacity. Similarly, as shown in Fig. 5B, the TP/(TP + FN) ratio, which represents the proportion of true positives (TP) among all positive predictions (TP + FN), is substantially higher for the ALL and Transformer models compared to the CNN model. This trend is most apparent during the first five epochs of training, where the ALL and Transformer models quickly achieve higher TP/(TP + FN) ratios. In contrast, the CNN model demonstrates slower progress in improving this ratio and plateaus at a later stage of training.

The models’ differences are also evident in the validation set performance (Fig. 5C). The ALL model achieves the highest F1 scores across all sequencing data, especially on CCS data. The transformer model performs similarly but lags slightly on CLR data. The CNN model, however, shows unstable performance with the lowest F1 scores, particularly on ONT data. In terms of the TP/(TP + FN) ratio on the validation set (Fig. 5D), the ALL model consistently achieves the highest ratio, demonstrating strong generalization across datasets. In contrast, the CNN model shows the lowest and most fluctuating TP/(TP + FN) ratio.

The results of the ablation experiments reveal that the complete ALL model, combining the CNN and Transformer modules, exhibits the best performance and stability in variant detection. The Transformer module plays a key role in enhancing the model’s performance, particularly excelling on CCS and ONT data, while the CNN module contributes less to the model’s stability. Overall, the performance of the ALL model on multiple sequencing datasets validates its robustness and broad applicability in SV detection, ensuring SVHunter’s efficiency and reliability across various types of genomic data.

## Discussion and conclusion

This study presents SVHunter, an SV detection tool for long-read sequencing, based on the transformer architecture. SVHunter combines CNNs and the Transformer architecture, which enables it to capture both local and global feature patterns, achieving high-precision detection of complex SVs. SVHunter directly processes raw feature matrices extracted from alignments. This ensures that nucleotide-level and alignment-level information is preserved throughout the detection process. By retaining these fine-grained details, SVHunter is able to more accurately identify subtle SV signals, including those that might be lost during data transformation in other methods. This design gives SVHunter a critical advantage in detecting complex or smaller SVs. Additionally, SVHunter employs the mean shift clustering algorithm, which dynamically adjusts to accommodate different types of SVs without requiring a pre-defined number of clusters, thereby accurately identifying breakpoints. SVHunter is designed to support five major SV types: deletions, INS, DUP, INV, and TRA. This broad coverage is achieved through the integration of CNNs for capturing local dependencies and Transformers for modeling long-range relationships, which enables SVHunter to generalize across diverse SV types. This flexibility makes it more versatile and applicable in comprehensive genomic studies compared to methods with narrower scopes. Extensive experiments on long-read sequencing platforms, such as PacBio and ONT, demonstrated that SVHunter excels under varying coverage conditions, outperforming most mainstream tools in detecting various SV types.

Compared with traditional SV detection methods, SVHunter offers three key advantages. First, heuristic algorithms often struggle to capture variant signals in complex regions. By utilizing both CNNs and transformer networks, SVHunter can capture local dependencies and long-range relationships simultaneously, enabling more effective handling of long-read data and reducing false-positive from repetitive regions (Supplementary Fig. S1). Second, SVHunter’s adoption of the mean shift clustering algorithm ensures precise breakpoint detection across different SV types. Traditional tools typically rely on manually designed rules or thresholds, which cannot automatically adapt to the complexity of different SV signals and are more susceptible to noise. In contrast, SVHunter achieves breakpoints that are closer to the true variant locations (Supplementary Fig. S2), indicating greater flexibility than heuristic-based methods. Third, SVHunter exhibits strong robustness against sequencing noise and varying coverage levels, performing exceptionally well across different sequencing platforms and coverage depths. This robustness is validated by the high F1 scores and low false-positive rates reported in the results section.

Despite its outstanding performance in various scenarios, SVHunter has certain limitations. First, because SVHunter employs a deep learning-based approach, the detection process involves both feature generation and model inference stages, which may result in a slightly slower runtime compared to traditional heuristic methods (Supplementary Table S15). Future research could focus on optimizing the model or adopting more efficient computational architectures to reduce computing costs and accelerate detection speed. Additionally, while SVHunter performs well under low coverage (e.g., 5x), its recall and precision are still lower than those under high-coverage conditions. Future work could further optimize the handling of low-coverage data to improve detection performance under extreme conditions. Finally, SVHunter cannot currently detect somatic and other complex SVs effectively. Specifically, it does not support the detection of nested SVs (e.g., INV within INV) or overlapping SVs between homologous chromosomes. The resolution of such complex SV typically requires additional information, which is beyond the current scope of SVHunter. Moreover, SVHunter has the potential to be extended for pan-genome-scale applications by incorporating prior knowledge from different species, enabling more comprehensive detection of SVs across diverse genomes. Future research may focus on enhancing SVHunter’s ability to handle such complex SVs.

While experimental methods such as Nanopore duplex sequencing or PCR amplification can improve sequencing accuracy by reducing error rates, SVHunter is designed to work effectively with standard long-read sequencing data without requiring additional experimental adaptations. By leveraging deep learning techniques, SVHunter inherently mitigates the impact of sequencing noise through its CNN and Transformer-based feature extraction and filtering strategies. This ensures that SVHunter remains broadly applicable across diverse sequencing conditions without necessitating specialized protocols. Future work could explore the integration of sequencing quality metrics into the model to further enhance its robustness to noisy data.

In conclusion, this study introduces SVHunter, an SV detection tool based on the transformer architecture, which combines local feature encoding with global dependency modeling. SVHunter has demonstrated excellent performance across various sequencing platforms and coverage conditions. It not only excels in detecting traditional SV types such as deletions and INS, but also has significant advantages in detecting other variants, such as INV and TRA. Through unsupervised clustering and automated read filtering strategies, SVHunter effectively reduces the number of false-positives, significantly improving detection accuracy and reliability. Although there are still some limitations to overcome—such as improving runtime efficiency and enhancing the detection of complex variants—SVHunter’s innovative design and broad applicability provide a new tool and approach for future genomic research and clinical applications.

**Key Points**

- SVHunter is a long-read SV detection tool based on the transformer architecture, that combines CNNs with transformer models to capture both local and global features simultaneously. This enables the tool to handle complex variant signals, significantly improving the accuracy in detecting complex structural variants.
- SVHunter introduces the mean shift clustering algorithm to accurately identify SV breakpoint locations. The mean shift clustering dynamically adjusts the bandwidth parameter according to different SV types, allowing for more flexible handling of variant signals in different genomic contexts, thereby increasing the precision of breakpoint detection.
- SVHunter employs an intelligent read selection and filtering strategy to ensure that all reliable reads are utilized to support SV detection. This strategy dynamically adjusts the minimum number of supporting reads required for each variant type based on factors such as global and local coverage differences and read error rates, preventing missed detections owing to inconsistent coverage.
- SVHunter integrates a voting mechanism from multiple methods to infer variant genotypes, combining techniques such as Bayesian inference, the expectation–maximization algorithm, and support ratios, ensuring higher accuracy in genotype detection.

## Supplementary Material

SVHunter_Supplementary_Materials_25_3_30_gao_bbaf203

## Data Availability

The sequencing data and ground truth call sets in this study are listed in Supplementary Table S19. The SVHunter code is available at https://github.com/eioyuou/SVHunter.

## References

[1] Liu YH, Luo C, Golding SG, et al. Tradeoffs in alignment and assembly-based methods for structural variant detection with long-read sequencing data. Nat Commun 2024;15:2447. 10.1038/s41467-024-46614-z. 38503752 PMC10951360

[2] Mills RE, Walter K, Stewart C, et al. Mapping copy number variation by population-scale genome sequencing. Nature 2011;470:59–65. 10.1038/nature09708. 21293372 PMC3077050

[3] Bennett EP, Petersen BL, Johansen IE, et al. INDEL detection, the ‘Achilles heel’of precise genome editing: A survey of methods for accurate profiling of gene editing induced indels. Nucleic Acids Res 2020;48:11958–81. 10.1093/nar/gkaa975. 33170255 PMC7708060

[4] Kim S, Misra A. SNP genotyping: Technologies and biomedical applications. Annu Rev Biomed 2007;9:289–320. 10.1146/annurev.bioeng.9.060906.152037 17391067

[5] Marshall CR, Noor A, Vincent JB, et al. Structural variation of chromosomes in autism spectrum disorder. The American Journal of Human Genetics 2008;82:477–88. 10.1016/j.ajhg.2007.12.009. 18252227 PMC2426913

[6] Sebat J, Levy DL, McCarthy SE. Rare structural variants in schizophrenia: One disorder, multiple mutations; one mutation, multiple disorders. Trends Genet 2009;25:528–35. 10.1016/j.tig.2009.10.004. 19883952 PMC3351381

[7] Raybould R, Sims R. Searching the dark genome for Alzheimer’s disease risk variants. Brain Sci 2021;11:332. 10.3390/brainsci11030332. 33800766 PMC7999247

[8] Slatko BE, Gardner AF, Ausubel FM. Overview of next-generation sequencing technologies. Curr Protoc Mol Biol 2018;122:e59. 10.1002/cpmb.59. 29851291 PMC6020069

[9] Lee H, Gurtowski J, Yoo S, et al. Third-generation sequencing and the future of genomics[J]. BioRxiv 2016;048603.

[10] Chen X, Schulz-Trieglaff O, Shaw R, et al. Manta: Rapid detection of structural variants and indels for germline and cancer sequencing applications. Bioinformatics 2016;32:1220–2. 10.1093/bioinformatics/btv710. 26647377

[11] Cameron DL, Baber J, Shale C, et al. GRIDSS2: Comprehensive characterisation of somatic structural variation using single breakend variants and structural variant phasing. Genome Biol 2021;22:1–25. 34253237 10.1186/s13059-021-02423-x PMC8274009

[12] Santuari L, Georgievska S, Kuzniar A, et al. Sv-channels: Filtering genomic deletions using one-dimensional convolutional neural networks. bioRxiv 2024;2024:17.618894.

[13] De Vree PJP, De Wit E, Yilmaz M, et al. Targeted sequencing by proximity ligation for comprehensive variant detection and local haplotyping. Nat Biotechnol 2014;32:1019–25. 10.1038/nbt.2959. 25129690

[14] Valle-Inclan JE, Besselink NJM, de Bruijn E, et al. A multi-platform reference for somatic structural variation detection[J]. Cell Genomics 2022;2. 10.1016/j.xgen.2022.100139. PMC9903816 36778136

[15] Mahmoud M, Gobet N, Cruz-Dávalos DI, et al. Structural variant calling: The long and the short of it. Genome Biol 2019;20:1–14. 31747936 10.1186/s13059-019-1828-7 PMC6868818

[16] Cameron DL, Di Stefano L, Papenfuss AT. Comprehensive evaluation and characterisation of short read general-purpose structural variant calling software. Nat Commun 2019;10:3240. 31324872 10.1038/s41467-019-11146-4 PMC6642177

[17] Rhoads A, Au KF. PacBio sequencing and its applications. Genomics, Proteomics and Bioinformatics 2015;13:278–89. 10.1016/j.gpb.2015.08.002. PMC4678779 26542840

[18] Lin B, Hui J, Mao H. Nanopore technology and its applications in gene sequencing. Biosensors 2021;11:214. 10.3390/bios11070214. 34208844 PMC8301755

[19] Amarasinghe SL, Su S, Dong X, et al. Opportunities and challenges in long-read sequencing data analysis. Genome Biol 2020;21:30. 10.1186/s13059-020-1935-5. 32033565 PMC7006217

[20] Logsdon GA, Vollger MR, Eichler EE. Long-read human genome sequencing and its applications. Nat Rev Genet 2020;21:597–614. 10.1038/s41576-020-0236-x. 32504078 PMC7877196

[21] Sedlazeck FJ, Rescheneder P, Smolka M, et al. Accurate detection of complex structural variations using single-molecule sequencing. Nat Methods 2018;15:461–8. 10.1038/s41592-018-0001-7. 29713083 PMC5990442

[22] Zhang Z, Jiang T, Li G, et al. Kled: An ultra-fast and sensitive structural variant detection tool for long-read sequencing data. Brief Bioinform 2024;25:bbae049. 10.1093/bib/bbae049. 38385878 PMC10883419

[23] Jiang T, Liu Y, Jiang Y, et al. Long-read-based human genomic structural variation detection with cuteSV. Genome Biol 2020;21:1–24. 10.1186/s13059-020-02107-y PMC7477834 32746918

[24] Smolka M, Paulin LF, Grochowski CM, et al. Detection of mosaic and population-level structural variants with Sniffles2[J]. Nature biotechnology 2024 42:1571–80. 10.1038/s41587-024-02141-2. PMC11217151 38168980

[25] Heller D, Vingron M. SVIM: Structural variant identification using mapped long reads. Bioinformatics 2019;35:2907–15. 10.1093/bioinformatics/btz041. 30668829 PMC6735718

[26] Gao R, Luo J, Ding H, et al. INSnet: A method for detecting insertions based on deep learning network. BMC bioinformatics 2023;24:80. 36879189 10.1186/s12859-023-05216-0 PMC9990265

[27] Lin J, Wang S, Audano PA, et al. SVision: A deep learning approach to resolve complex structural variants. Nat Methods 2022;19:1230–3. 10.1038/s41592-022-01609-w. 36109679 PMC9985066

[28] Ding H, Luo J. MAMnet: Detecting and genotyping deletions and insertions based on long reads and a deep learning approach. Brief Bioinform 2022;23:bbac195. 10.1093/bib/bbac195. 35580841

[29] Kattenborn T, Leitloff J, Schiefer F, et al. Review on convolutional neural networks (CNN) in vegetation remote sensing. ISPRS journal of photogrammetry and remote sensing 2021;173:24–49. 10.1016/j.isprsjprs.2020.12.010.

[30] Han K, Wang Y, Chen H, et al. A survey on vision transformer. IEEE Trans Pattern Anal Mach Intell 2022;45:87–110. 10.1109/TPAMI.2022.3152247. 35180075

[31] Comaniciu D, Meer P. Mean shift: A robust approach toward feature space analysis. IEEE Trans Pattern Anal Mach Intell 2002;24:603–19. 10.1109/34.1000236.

[32] Woo S, Park J, Lee JY, et al. Cbam: Convolutional Block Attention Module[C]//Proceedings of the European Conference on Computer Vision (ECCV), 2018, 3–19.

[33] Indolia S, Goswami AK, Mishra SP, et al. Conceptual understanding of convolutional neural network-a deep learning approach. Procedia computer science 2018;132:679–88. 10.1016/j.procs.2018.05.069.

[34] Karita S, Chen N, Hayashi T, et al. A comparative study on transformer vs rnn in speech applications[C]//2019 IEEE automatic speech recognition and understanding workshop (ASRU). IEEE 2019;449–56.

[35] Zhao L, Ji S. CNN, RNN, or ViT? An evaluation of different deep learning architectures for spatio-temporal representation of sentinel time series. IEEE Journal of Selected Topics in Applied Earth Observations and Remote Sensing 2022;16:44–56.

[36] van de Schoot R, Depaoli S, King R, et al. Bayesian statistics and modelling. Nature Reviews Methods Primers 2021;1:1. 10.1038/s43586-020-00001-2.

[37] Moon TK . The expectation-maximization algorithm. IEEE Signal Process Mag 1996;13:47–60. 10.1109/79.543975.

[38] Zook JM, Hansen NF, Olson ND, et al. A robust benchmark for detection of germline large deletions and insertions. Nat Biotechnol 2020;38:1347–55. 10.1038/s41587-020-0538-8. 32541955 PMC8454654

[39] Jeffares DC, Jolly C, Hoti M, et al. Transient structural variations have strong effects on quantitative traits and reproductive isolation in fission yeast. Nat Commun 2017;8:14061. 10.1038/ncomms14061. 28117401 PMC5286201

[40] Ono Y, Asai K, Hamada M. PBSIM2: A simulator for long-read sequencers with a novel generative model of quality scores. Bioinformatics 2021;37:589–95. 10.1093/bioinformatics/btaa835. 32976553 PMC8097687

[41] Rhie A, Nurk S, Cechova M, et al. The complete sequence of a human Y chromosome. Nature 2023;621:344–54. 10.1038/s41586-023-06457-y. 37612512 PMC10752217

[42] Garg S, Fungtammasan A, Carroll A, et al. Chromosome-scale, haplotype-resolved assembly of human genomes. Nat Biotechnol 2021;39:309–12. 10.1038/s41587-020-0711-0. 33288905 PMC7954703

[43] Shafin K, Pesout T, Lorig-Roach R, et al. Nanopore sequencing and the Shasta toolkit enable efficient de novo assembly of eleven human genomes. Nat Biotechnol 2020;38:1044–53. 10.1038/s41587-020-0503-6. 32686750 PMC7483855

[44] Li H, Bloom JM, Farjoun Y, et al. A synthetic-diploid benchmark for accurate variant-calling evaluation. Nat Methods 2018;15:595–7. 10.1038/s41592-018-0054-7. 30013044 PMC6341484

[45] Kim YE, Ki CS, Jang MA. Challenges and considerations in sequence variant interpretation for mendelian disorders[J]. Annals of Laboratory Medicine 2019;39:421. 10.3343/alm.2019.39.5.421 PMC6502951 31037860

