# Correction to: SVHunter: long-read-based structural variation detection through the transformer model

**DOI:** 10.1093/bib/bbag221

**Published:** 2026-04-22

This is a correction to: Runtian Gao, Heng Hu, Zhongjun Jiang, Shuqi Cao, Guohua Wang, Yuming Zhao, Tao Jiang, SVHunter: long-read-based structural variation detection through the transformer model, *Briefings in Bioinformatics*, Volume 26, Issue 3, May 2025, bbaf203, https://doi.org/10.1093/bib/bbaf203

Corresponding authorship has been changed on this paper to be solely Tao Jiang.

