# Supplementary material for: SVHunter: long-read-based structural variation detection through the transformer model
Source: Brief Bioinform. 2025 May 9;26(3):bbaf203. doi: 10.1093/bib/bbaf203 (PMC12062572; doi:10.1093/bib/bbaf203)
Supplement: SVHunter_Supplementary_Materials_25_3_30_gao_bbaf203 [file svhunter_supplementary_materials_25_3_30_gao_bbaf203.docx]

**SVHunter: long-read-based structural variation detection through transformer model**

**Supplementary Materials**

**Content**

[Figure S1. Results of the alignment between CHM13 and its own reference genome 4](#_Toc191045253)

[Figure S2. An example of a large insertion only reported by SVHunter successfully 5](#_Toc191045254)

[Figure S3. The structure of the CNN Encoder module 6](#_Toc191045255)

[Figure S4 Comparison of validation loss curves of different models (first 20 epochs) 7](#_Toc191045256)

[Figure S5 Comparison of validation loss curves of different models (early stopping) 8](#_Toc191045257)

[Figure S6 Comparison of test loss curves of different models (first 20 epochs) 9](#_Toc191045258)

[Figure S7 Comparison of test loss curves of different models (early stopping) 10](#_Toc191045259)

[Figure S8 Evaluating test F1 score variability across models: first 20 epochs vs. early stopping 11](#_Toc191045260)

[Figure S9 Ablation study results comparing the performance of different model configurations across sequencing platforms (early stopping) 12](#_Toc191045261)

[Figure S10 Mean F1-score comparison across tools on five independent simulated datasets 13](#_Toc191045262)

[Table S1 Benchmark results on HG002 SV callsets 14](#_Toc191045263)

[Table S2 Benchmark results on HG002 genotyping-SV callsets 15](#_Toc191045264)

[Table S3 Benchmark results of short read and long read tools on HG002 High-Confidence Regions 16](#_Toc191045265)

[Table S4 Benchmark results of short read and long read tools on HG002 Non-Repetitive High-Confidence Regions 17](#_Toc191045266)

[Table S5 Benchmark results on simulated illumina data 18](#_Toc191045267)

[Table S6 Benchmark results on simulated ONT data 19](#_Toc191045268)

[Table S7 Benchmark results on simulated CLR data 20](#_Toc191045269)

[Table S8 Benchmark results on simulated CLR data with the same SV type count 21](#_Toc191045270)

[Table S9 Benchmark results on HG002 for SV callsets of dfifferent lengths 22](#_Toc191045271)

[Table S10 Benchmark results on CHM13 CLR data 23](#_Toc191045272)

[Table S11 Benchmark results on CHM13 CLR data with different coverage 24](#_Toc191045273)

[Table S12 The false discovered SV records on CHM13 data with different coverage 25](#_Toc191045274)

[Table S13 Results on mendelian discordance rate for HG002-HG004 26](#_Toc191045275)

[Table S14 Results on mendelian discordance rate for HG005-HG007 27](#_Toc191045276)

[Table S15 Runtime and memory usage in 28× HG002 HiFi dataset 28](#_Toc191045277)

[Table S16 Benchmark results on A.thaliana using simulated dataset 29](#_Toc191045278)

[Table S17 Benchmark results for A.thaliana across five independent simulated datasets 30](#_Toc191045279)

[Table S18 Benchmark results on A.thaliana using real dataset 31](#_Toc191045280)

[Table S19 Data availability 32](#_Toc191045281)

[Supplementary Notes 33](#_Toc191045282)

[1. Feature Extraction 33](#_Toc191045283)

[2. Model train 33](#_Toc191045284)

[3. Evaluation of SV calling 34](#_Toc191045285)

[4. Additional considerations in CHM13 benchmarking 35](#_Toc191045286)

[5. Commands used for benchmark 40](#_Toc191045287)


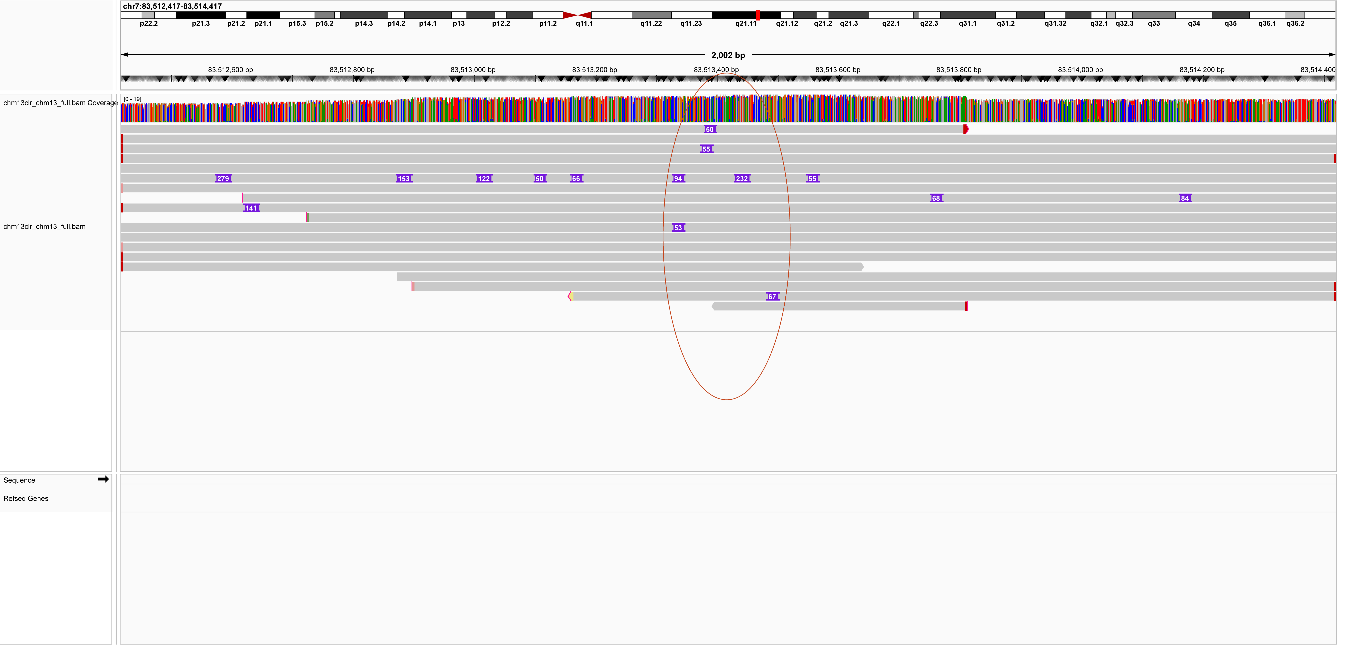


**Figure S1. Results of the alignment between CHM13 and its own reference genome**

Theoretically, there are no variants should be present. Only SVHunter successfully filtered out false positives. The IGV screenshot of the CHM13 data (with INDELs smaller than 50bp hidden) shows multiple insertional noise events of varying sizes in this region. SVIM, Sniffles2, and cuteSV reported structural variants of 53bp, 55bp, and 64bp, respectively.


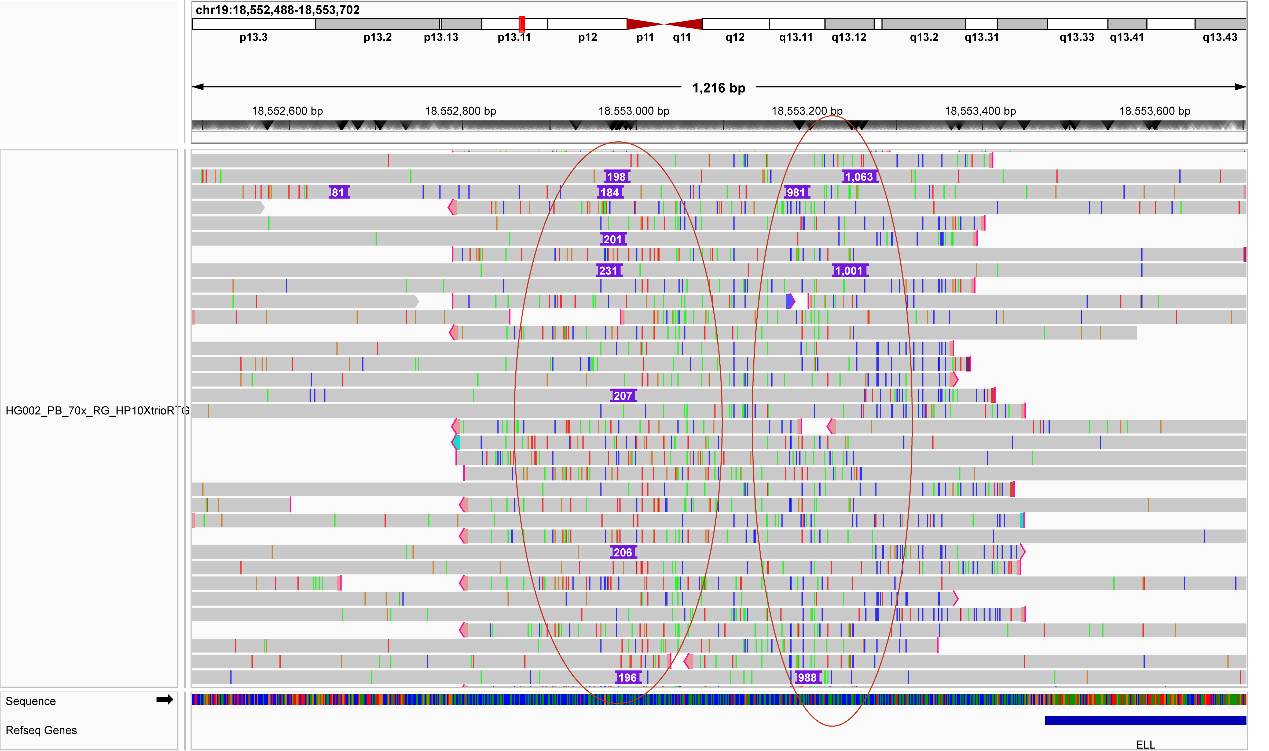


**Figure S2. An example of a large insertion only reported by SVHunter successfully**

There is an 1191bp on the chromosome 19 on the HG002 ground truth set. The IGV screenshot of the CLR 69X data (with INDELs smaller than 50bp hidden) shows that SVHunter, using flexible Mean Shift clustering, reported a 988bp SV within the error margin. In contrast, cuteSV failed to detect this SV, while Sniffles2 and SVIM reported SVs of 226bp and 565bp, respectively, but these exceeded the call error due to insufficient clustering precision.


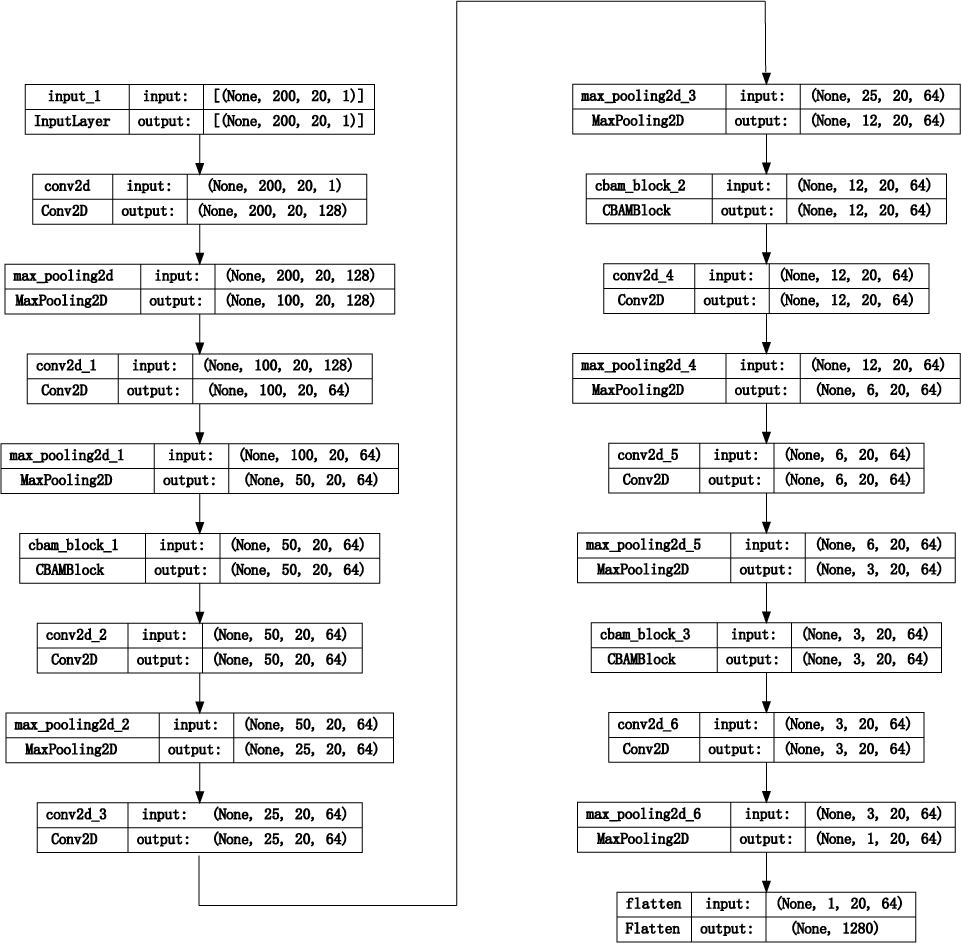


**Figure S3. The structure of the CNN Encoder module**

**
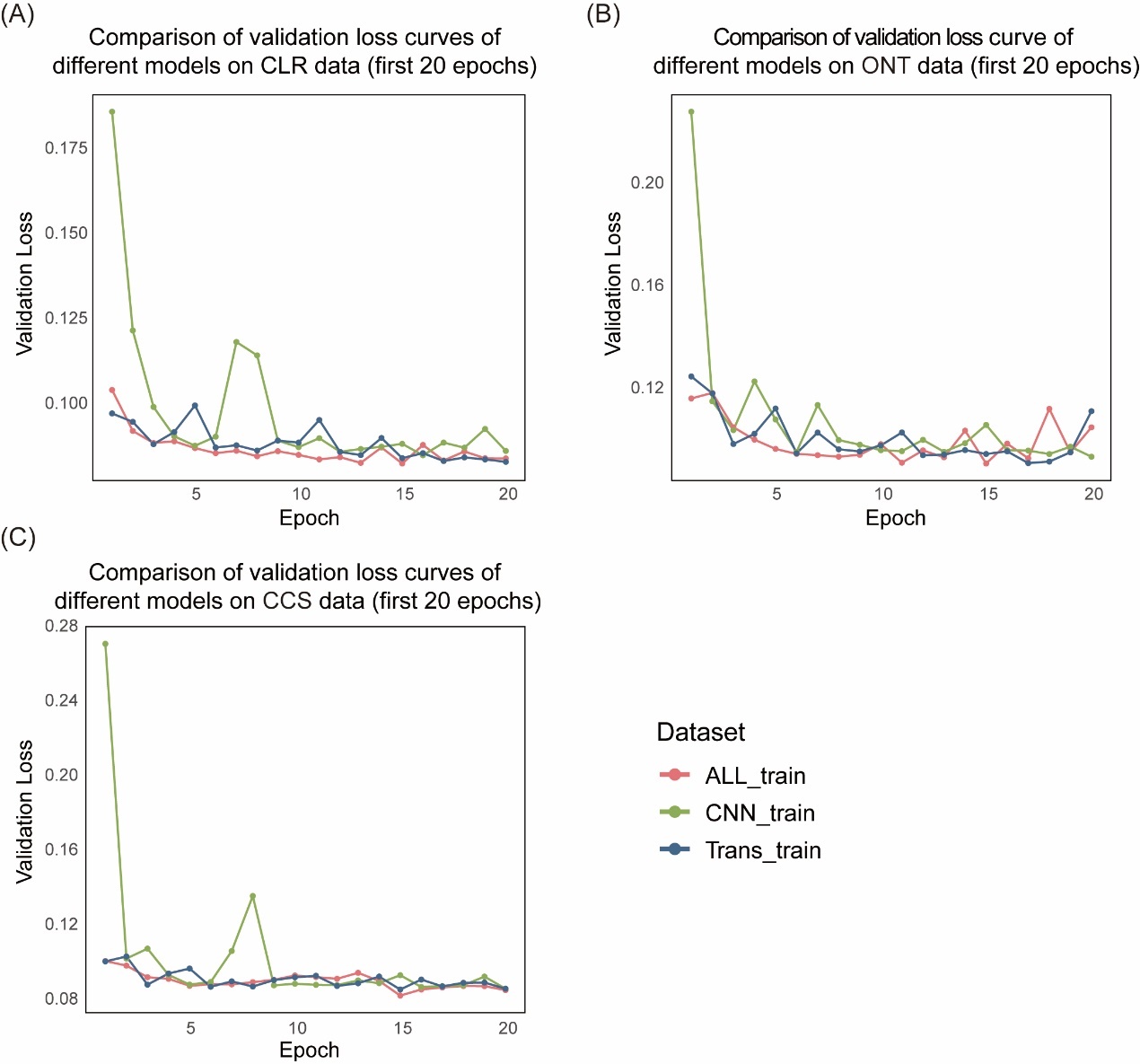
**

**Figure S4 Comparison of validation loss curves of different models (first 20 epochs)**

**
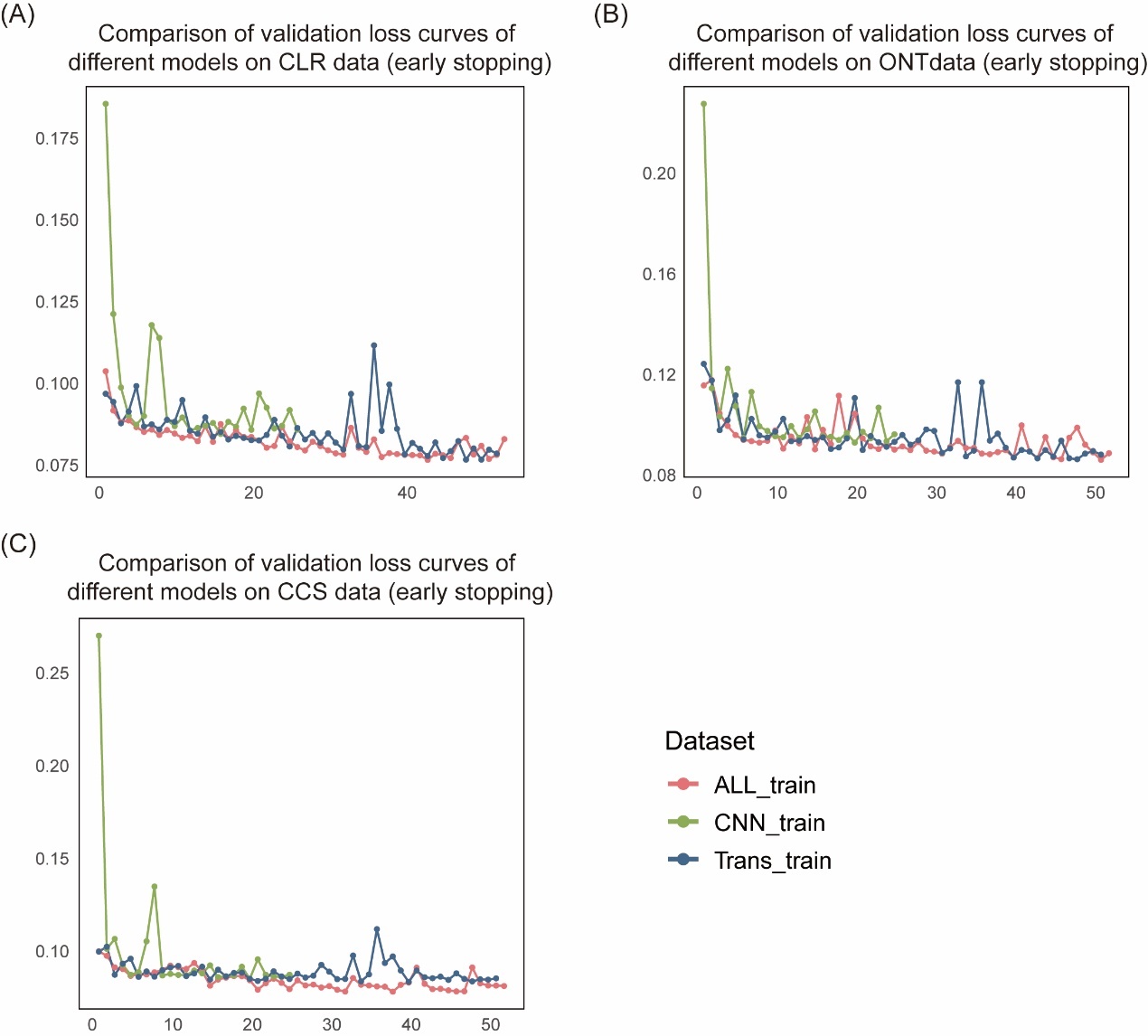
**

**Figure S5 Comparison of validation loss curves of different models (early stopping)**

**
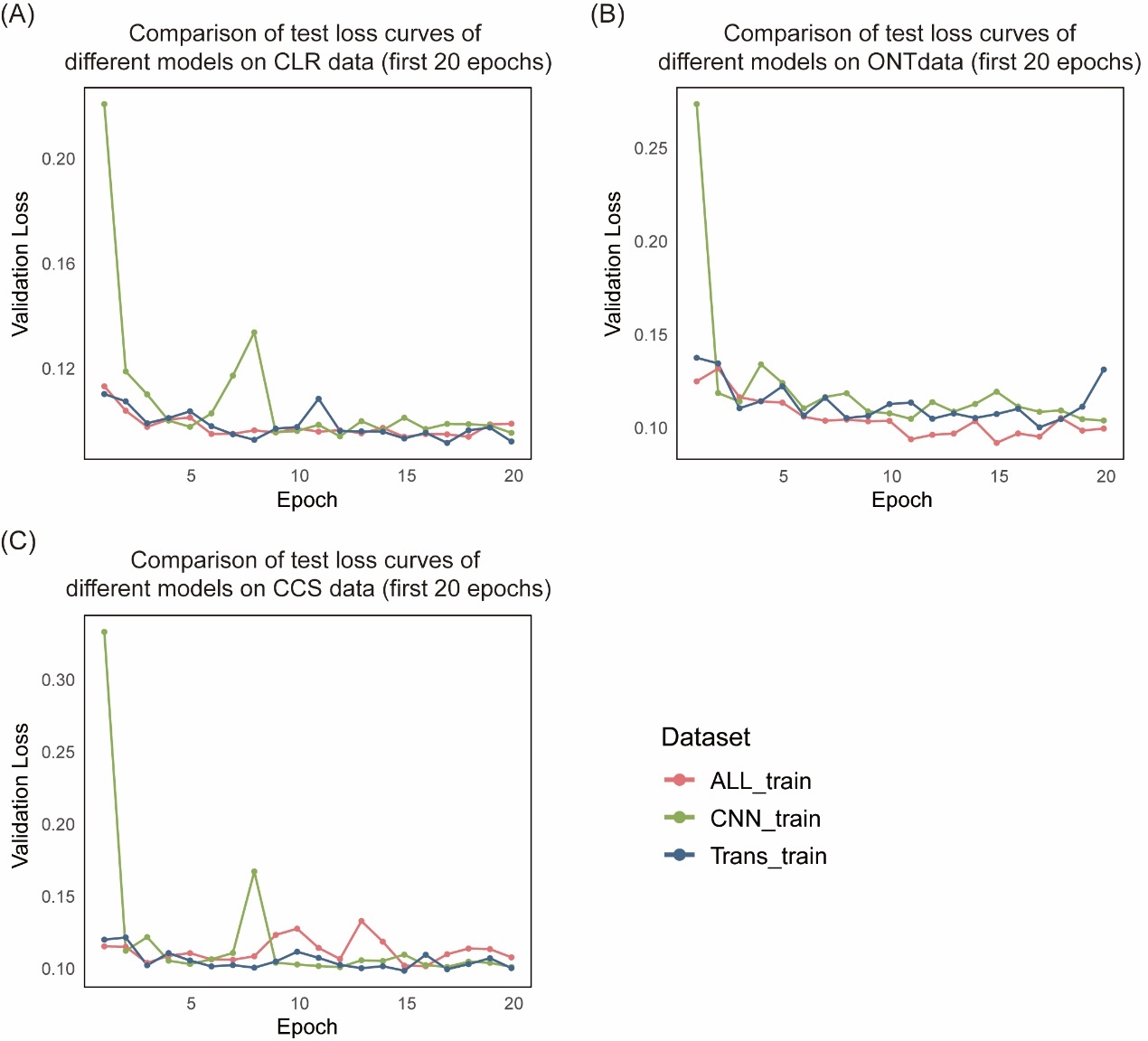
**

**Figure S6 Comparison of test loss curves of different models (first 20 epochs)**

**
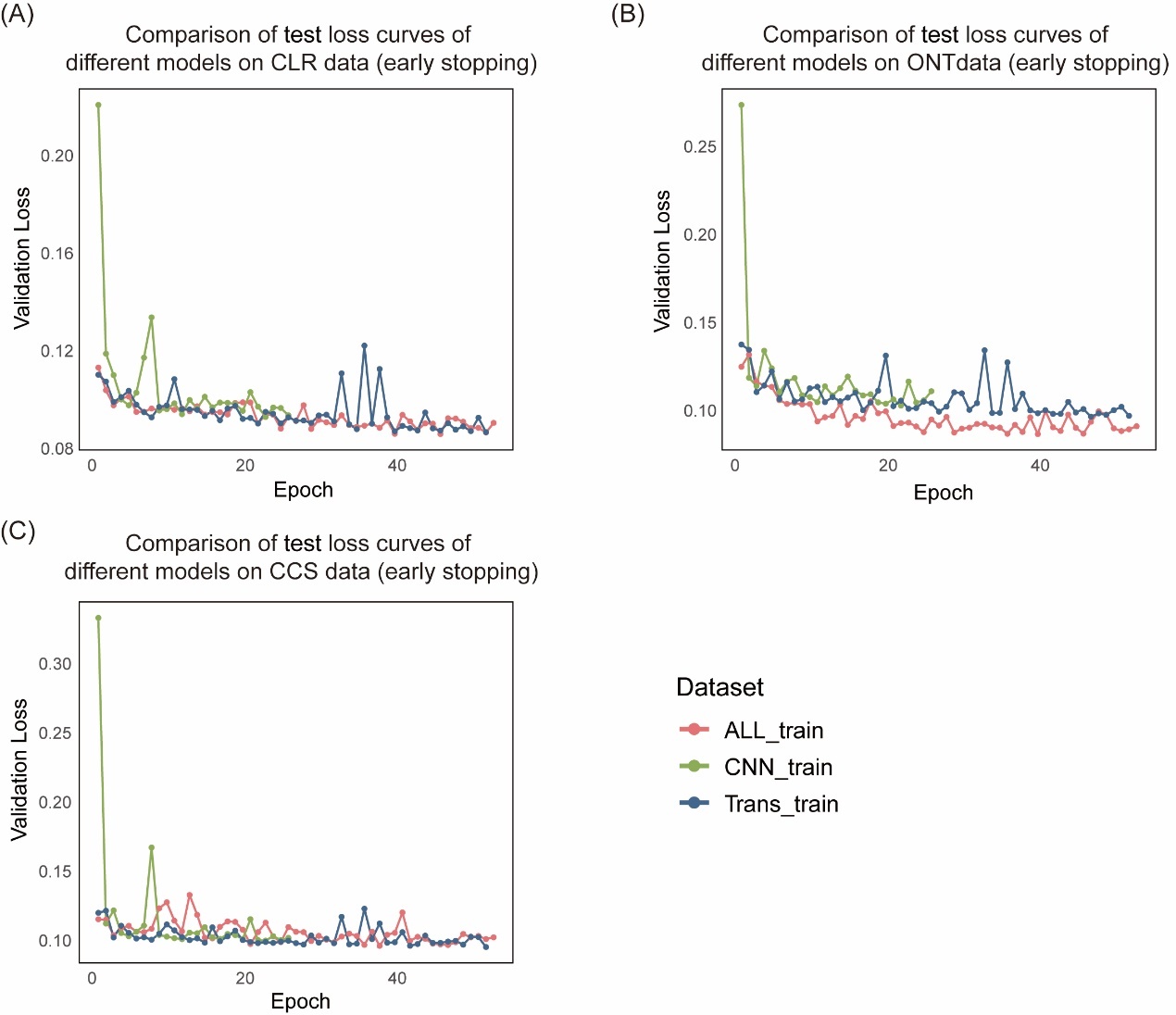
**

**Figure S7 Comparison of test loss curves of different models (early stopping)**

**
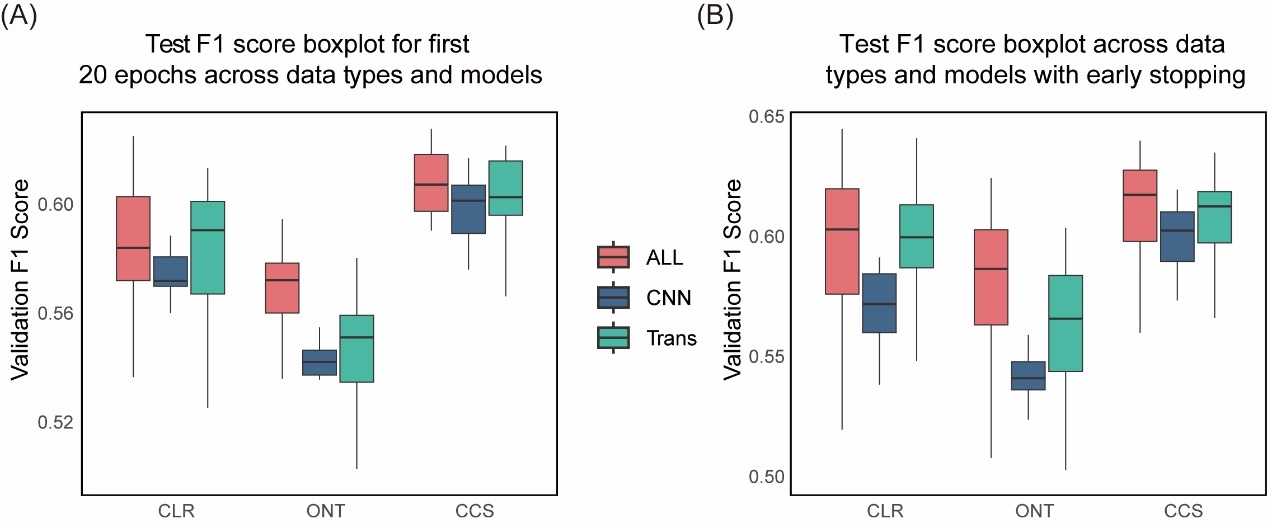
**

**Figure S8 Evaluating test F1 score variability across models: first 20 epochs vs. early stopping**

**
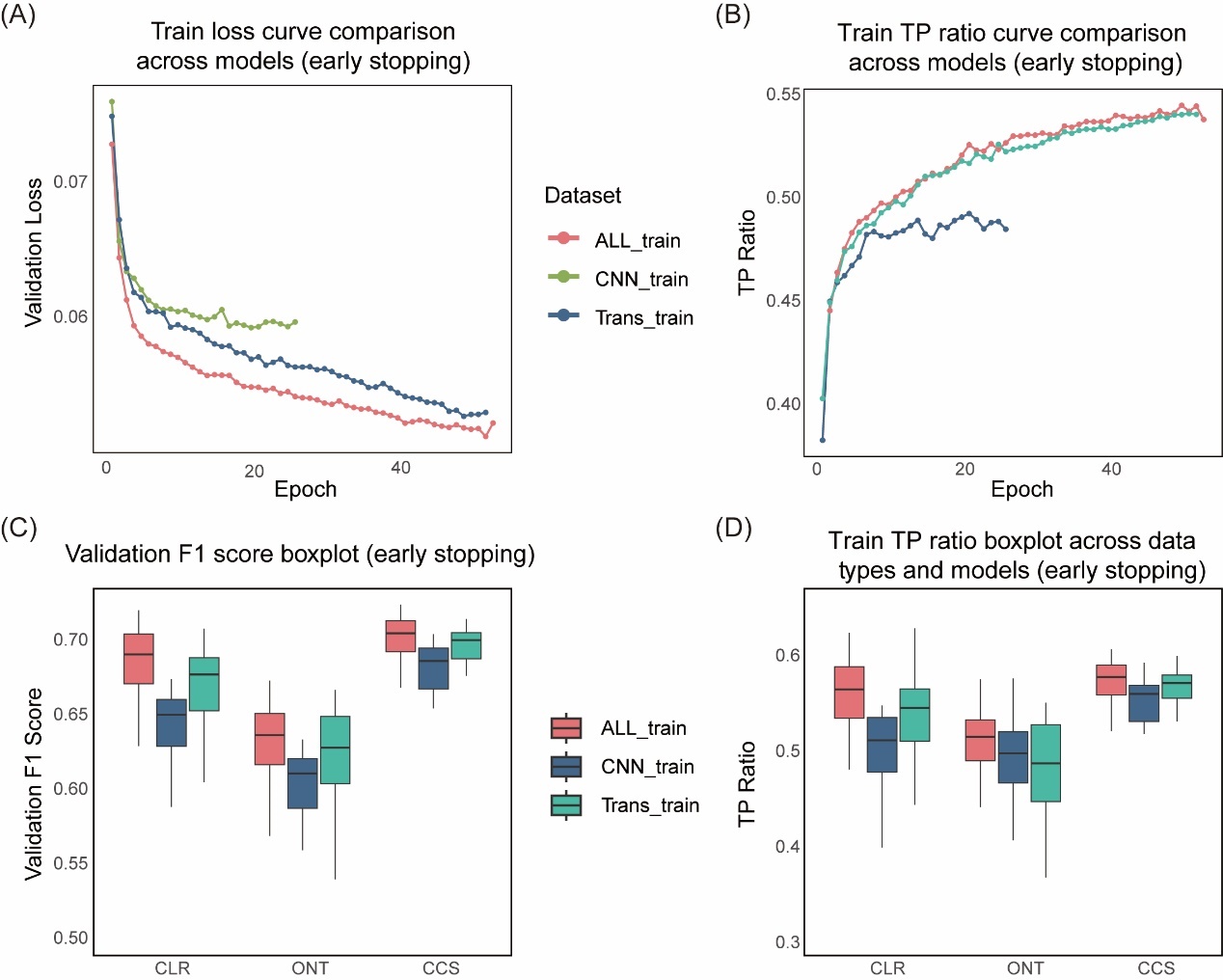
**

**Figure S9 Ablation study results comparing the performance of different model configurations across sequencing platforms (early stopping)**

**
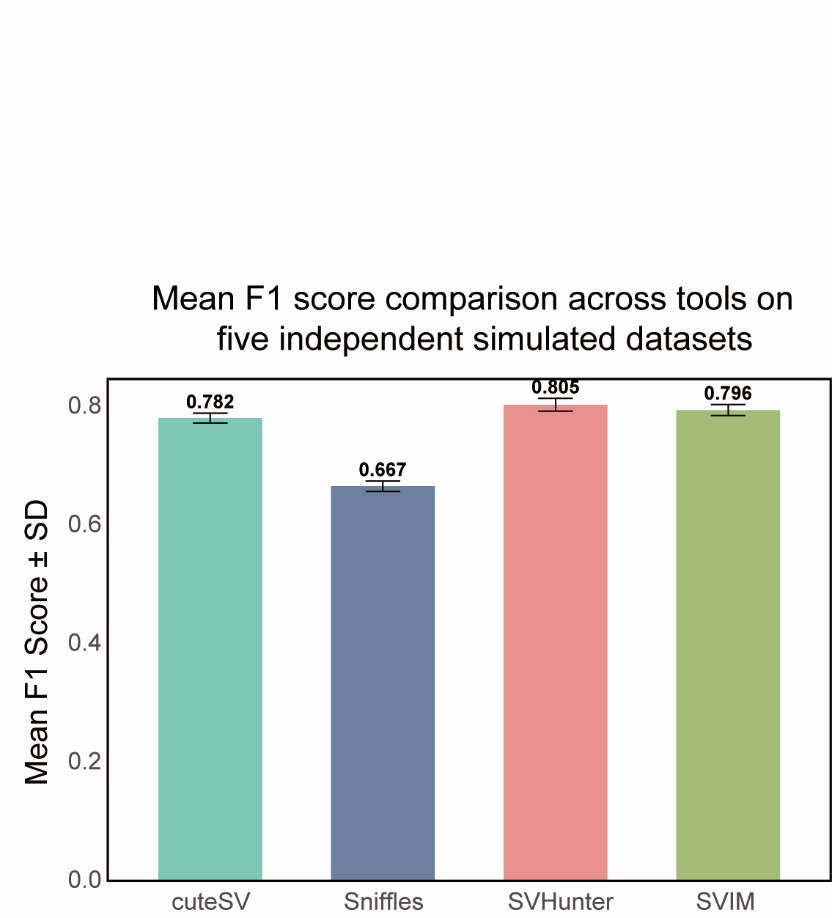
**

**Figure S10 Mean F1-score comparison across tools on five independent simulated datasets**

**Table S1 Benchmark results on HG002 SV callsets**

| **Dataset** | **Tool** | **INS** | | | **DEL** | | |
| --- | --- | --- | --- | --- | --- | --- | --- |
|  |  | **Precision** | **Recall** | **F1** | **Precision** | **Recall** | **F1** |
| PacBio  CLR 69× | Sniffles2 | 0.6928 | 0.8698 | 0.7713 | 0.9702 | 0.9481 | 0.959 |
|  | SVIM | **0.9479** | 0.7921 | 0.863 | 0.9698 | 0.9367 | 0.953 |
|  | cuteSV | 0.9447 | 0.8802 | 0.9113 | **0.9781** | 0.9197 | 0.948 |
|  | SVHunter | 0.9406 | **0.8931** | **0.9162** | 0.9727 | **0.9623** | **0.9675** |
| PacBio  CLR 35× | Sniffles2 | 0.7517 | 0.8589 | 0.8018 | **0.974** | 0.9311 | 0.952 |
|  | SVIM | 0.9378 | 0.798 | 0.8623 | 0.9668 | 0.9325 | 0.9493 |
|  | cuteSV | 0.943 | 0.8599 | 0.8995 | 0.9551 | 0.8954 | 0.9243 |
|  | SVHunter | **0.9464** | **0.8658** | **0.9043** | 0.973 | **0.9467** | **0.9597** |
| PacBio  CLR 20× | Sniffles2 | 0.7055 | **0.8252** | 0.7607 | 0.9717 | 0.9026 | 0.9359 |
|  | SVIM | 0.9343 | 0.7396 | 0.8256 | 0.9691 | 0.8927 | 0.9293 |
|  | cuteSV | 0.9451 | 0.7931 | 0.8624 | **0.98** | 0.8685 | 0.9209 |
|  | SVHunter | **0.9388** | 0.8208 | **0.8759** | 0.9624 | **0.9275** | **0.9446** |
| PacBio  CLR 10× | Sniffles2 | 0.5039 | **0.7347** | 0.5978 | 0.9646 | 0.8323 | 0.8936 |
|  | SVIM | 0.9255 | 0.5906 | 0.7211 | 0.9772 | 0.7619 | 0.8562 |
|  | cuteSV | **0.943** | 0.6465 | 0.7671 | **0.9784** | 0.7391 | 0.8421 |
|  | SVHunter | 0.9348 | 0.6599 | **0.7737** | 0.9617 | **0.8564** | **0.906** |
| PacBio  CLR 5× | Sniffles2 | 0.457 | 0.5267 | 0.4894 | 0.9655 | 0.6162 | 0.7523 |
|  | SVIM | 0.5377 | 0.4906 | 0.5131 | 0.9634 | 0.6361 | 0.7663 |
|  | cuteSV | 0.6818 | 0.5282 | 0.5953 | **0.9689** | 0.6197 | 0.756 |
|  | SVHunter | **0.7683** | 0.4926 | **0.6003** | 0.8867 | **0.7342** | **0.8033** |
| PacBio  CCS 30× | Sniffles2 | 0.9068 | **0.9292** | 0.9178 | 0.9519 | 0.9417 | 0.9468 |
|  | SVIM | 0.8772 | 0.9124 | 0.8944 | 0.9486 | 0.9439 | 0.9462 |
|  | cuteSV | 0.8907 | 0.9243 | 0.9072 | **0.9636** | 0.9232 | 0.943 |
|  | SVHunter | **0.9189** | 0.9193 | **0.9191** | 0.9603 | **0.9446** | **0.9523** |
| PacBio  CCS 10× | Sniffles2 | 0.9034 | **0.8752** | 0.8891 | 0.9457 | 0.8913 | 0.9177 |
|  | SVIM | 0.861 | 0.8649 | 0.8329 | 0.9376 | **0.9183** | 0.9278 |
|  | cuteSV | 0.8978 | 0.8525 | 0.8746 | 0.9617 | 0.8571 | 0.9064 |
|  | SVHunter | **0.9229** | 0.8653 | **0.8932** | **0.9627** | **0.9183** | **0.94** |
| PacBio  CCS 5× | Sniffles2 | **0.913** | 0.7069 | 0.7969 | 0.9545 | 0.7 | 0.8077 |
|  | SVIM | 0.8099 | 0.848 | 0.8285 | **0.9164** | **0.8955** | 0.9058 |
|  | cuteSV | 0.8856 | **0.7941** | 0.8374 | 0.9602 | 0.7719 | 0.8558 |
|  | SVHunter | 0.9095 | 0.7911 | **0.8462** | 0.9616 | 0.8714 | **0.9142** |
| Oxford  Nanopore 50× | Sniffles2 | **0.8817** | 0.8777 | 0.8797 | 0.9117 | **0.9538** | 0.9323 |
|  | SVIM | 0.7963 | 0.8203 | 0.8081 | 0.914 | 0.9439 | 0.9287 |
|  | cuteSV | 0.8813 | 0.8416 | 0.861 | **0.9505** | 0.9289 | **0.9396** |
|  | SVHunter | 0.8657 | **0.9035** | **0.8842** | 0.8974 | 0.9694 | 0.932 |
| Oxford  Nanopore 20× | Sniffles2 | 0.87 | 0.8713 | 0.8706 | 0.8815 | 0.941 | 0.9103 |
|  | SVIM | 0.706 | 0.8168 | 0.7574 | 0.88 | 0.9382 | 0.9082 |
|  | cuteSV | **0.8851** | 0.8391 | 0.8615 | **0.941** | 0.9183 | **0.9295** |
|  | SVHunter | 0.8654 | **0.8782** | **0.8717** | 0.8747 | **0.9623** | 0.9164 |
| Oxford  Nanopore 10× | Sniffles2 | 0.863 | **0.8292** | **0.8457** | 0.8576 | 0.8905 | 0.8738 |
|  | SVIM | 0.7261 | 0.7233 | 0.7247 | 0.8703 | 0.8536 | 0.8619 |
|  | cuteSV | **0.8845** | 0.7579 | 0.8163 | **0.9188** | 0.8365 | 0.8757 |
|  | SVHunter | 0.8696 | 0.7921 | 0.829 | 0.8604 | **0.8977** | **0.8786** |
| Oxford  Nanopore 5× | Sniffles2 | 0.8627 | 0.6812 | 0.7613 | 0.8689 | 0.7257 | 0.7909 |
|  | SVIM | 0.6927 | 0.6272 | 0.6584 | 0.8217 | 0.7306 | 0.7735 |
|  | cuteSV | 0.8585 | 0.652 | 0.7411 | **0.8765** | 0.7214 | **0.7914** |
|  | SVHunter | **0.8634** | **0.6851** | **0.764** | 0.7803 | **0.747** | 0.7633 |

**Table S2 Benchmark results on HG002 genotyping-SV callsets**

| **Dataset** | **Tool** | **INS** | | | **DEL** | | |
| --- | --- | --- | --- | --- | --- | --- | --- |
|  |  | **Precision** | **Recall** | **F1** | **Precision** | **Recall** | **F1** |
| PacBio  CLR 69× | Sniffles2 | 0.6483 | 0.8621 | 0.74 | 0.9287 | 0.9459 | 0.9372 |
|  | SVIM | 0.7725 | 0.7564 | 0.7644 | 0.9249 | 9339 | 0.9294 |
|  | cuteSV | **0.9198** | 0.8773 | 0.8981 | **0.9599** | 0.9183 | 0.9387 |
|  | SVHunter | 0.9171 | **0.8906** | **0.9037** | 0.9195 | **0.9602** | **0.9394** |
| PacBio  CLR 35× | Sniffles2 | 0.6919 | 0.8486 | 0.7623 | 0.9249 | 0.9277 | 0.9263 |
|  | SVIM | 0.7661 | 0.7635 | 0.7648 | 0.9123 | 0.9287 | 0.9204 |
|  | cuteSV | **0.9153** | 0.8563 | 0.8848 | **0.9551** | 0.8954 | 0.9243 |
|  | SVHunter | 0.9129 | **0.8616** | **0.8865** | 0.9189 | **0.9437** | **0.9312** |
| PacBio  CLR 20× | Sniffles2 | 0.6386 | 0.8104 | 0.7143 | 0.9059 | 0.8963 | 0.901 |
|  | SVIM | 0.7786 | 0.703 | 7389 | 0.9144 | 0.887 | 0.9005 |
|  | cuteSV | **0.8944** | 0.7839 | 0.8355 | **0.9423** | 0.864 | 0.9014 |
|  | SVHunter | 0.8873 | **0.8123** | **0.8482** | 0.8938 | **0.9224** | **0.9079** |
| PacBio  CLR 10× | Sniffles2 | 0.4346 | 0.7048 | 0.5377 | 0.855 | 0.8148 | **0.8344** |
|  | SVIM | 0.7199 | 0.5288 | 0.6097 | 0.8624 | 0.7385 | 0.7956 |
|  | cuteSV | 0.8282 | 0.6163 | 0.7067 | **0.889** | 0.7203 | 0.7958 |
|  | SVHunter | **0.8555** | **0.6397** | **0.7321** | 0.814 | **0.8347** | 0.8242 |
| PacBio  CLR 5× | Sniffles2 | 0.3819 | 0.4818 | 0.4261 | 0.7873 | 0.567 | 0.6592 |
|  | SVIM | 0.2149 | 0.2779 | 0.2424 | 0.4769 | 0.4639 | 0.4703 |
|  | cuteSV | 0.5706 | **0.4837** | 0.5236 | **0.8044** | 0.5751 | 0.6707 |
|  | SVHunter | **0.661** | 0.4551 | **0.539** | 0.7382 | **0.6969** | **0.717** |
| PacBio  CCS 30× | Sniffles2 | 0.8729 | **0.9267** | 0.899 | 0.9289 | 0.9404 | 0.9346 |
|  | SVIM | 0.8115 | 0.906 | 0.8561 | 0.9279 | 0.9427 | 0.9352 |
|  | cuteSV | 0.8678 | 0.9224 | 0.8943 | **0.9414** | 0.9216 | 0.9314 |
|  | SVHunter | **0.9119** | 0.9187 | **0.9153** | 0.9386 | **0.9434** | **0.941** |
| PacBio  CCS 10× | Sniffles2 | 0.8459 | **0.8679** | 0.8566 | 0.88 | 0.8841 | 0.8821 |
|  | SVIM | 0.7141 | 0.8415 | 0.7726 | 0.7925 | 0.9047 | 0.8449 |
|  | cuteSV | 0.8295 | 0.8422 | 0.8358 | 0.8915 | 0.8476 | 0.869 |
|  | SVHunter | **0.8955** | 0.8618 | **0.8783** | **0.9232** | **0.9151** | **0.9191** |
| PacBio  CCS 5× | Sniffles2 | **0.8299** | 0.6868 | 0.7516 | 0.8353 | 0.6713 | 0.7444 |
|  | SVIM | 0.3404 | 0.7011 | 0.4583 | 0.3862 | 0.7832 | 0.5173 |
|  | cuteSV | 0.7764 | **0.7717** | 0.774 | 0.8037 | 0.739 | 0.77 |
|  | SVHunter | 0.8019 | 0.7695 | **0.7854** | **0.8824** | **0.8614** | **0.8718** |
| Oxford  Nanopore 50× | Sniffles2 | 0.8215 | 0.8699 | 0.845 | 0.8927 | 0.9529 | 0.9218 |
|  | SVIM | 0.6209 | 0.7807 | 0.6916 | 0.8906 | 0.9425 | 0.9158 |
|  | cuteSV | **0.8673** | 0.8394 | **0.8531** | **0.9389** | 9281 | **0.9335** |
|  | SVHunter | 0.8055 | **0.897** | 0.8488 | 0.8711 | **0.9685** | 0.9172 |
| Oxford  Nanopore 20× | Sniffles2 | 0.7949 | 0.8608 | 0.8265 | 0.8575 | 0.9395 | 0.8966 |
|  | SVIM | 0.5524 | 0.7772 | 0.6458 | 0.8567 | 0.9366 | 0.8948 |
|  | cuteSV | **0.8454** | 0.8328 | **0.8391** | **0.9192** | 0.9165 | **0.9178** |
|  | SVHunter | 0.799 | **0.8694** | 0.8327 | 0.843 | **0.961** | 0.8981 |
| Oxford  Nanopore 10× | Sniffles2 | 0.7486 | **0.8081** | 0.7772 | 0.8104 | 0.8849 | **0.846** |
|  | SVIM | 0.5556 | 0.6666 | 0.6061 | **0.8203** | 0.846 | 0.833 |
|  | cuteSV | **0.7984** | 0.7386 | 0.7674 | 0.861 | 0.8275 | 0.8439 |
|  | SVHunter | 0.7826 | 0.7742 | **0.7784** | 0.8018 | **0.891** | 0.844 |
| Oxford  Nanopore 5× | Sniffles2 | 0.6978 | 0.6334 | 0.6641 | **0.7617** | 0.6987 | **0.7288** |
|  | SVIM | 0.3368 | 0.4499 | 0.3852 | 0.4868 | 0.6164 | 0.544 |
|  | cuteSV | 0.7099 | 0.6077 | 0.6548 | 0.7453 | 0.6876 | 0.7153 |
|  | SVHunter | **0.7505** | **0.6542** | **0.699** | 0.7045 | **0.7272** | 0.7157 |

**Table S3 Benchmark results of short read and long read tools on HG002 High-Confidence Regions**

| **Dataset** | **Tool** | **INS** | | | **DEL** | | |
| --- | --- | --- | --- | --- | --- | --- | --- |
|  |  | **Precision** | **Recall** | **F1** | **Precision** | **Recall** | **F1** |
| PacBio  CLR 69×  VS  Illumina  70× | Sniffles2 | 0.6928 | 0.8698 | 0.7713 | 0.9702 | 0.9481 | 0.959 |
|  | SVIM | 0.9479 | 0.7921 | 0.863 | 0.9698 | 0.9367 | 0.953 |
|  | cuteSV | 0.9447 | 0.8802 | 0.9113 | 0.9781 | 0.9197 | 0.948 |
|  | **SVHunter** | **0.9406** | **0.8931** | **0.9162** | **0.9727** | **0.9623** | **0.9675** |
|  | Manta | 0.9405 | 0.2114 | 0.3452 | 0.8919 | 0.5686 | 0.6944 |
|  | GRIDSS | 0.9855 | 0.1 | 0.1832 | 0.9256 | 0.6013 | 0.729 |
|  | sv-channels | \ | \ | \ | 0.9035 | 0.5657 | 0.6958 |

**Table S4 Benchmark results of short read and long read tools on HG002 Non-Repetitive High-Confidence Regions**

| **Dataset** | **Tool** | **INS** | | | **DEL** | | |
| --- | --- | --- | --- | --- | --- | --- | --- |
|  |  | **Precision** | **Recall** | **F1** | **Precision** | **Recall** | **F1** |
| PacBio  CLR 69×  VS  Illumina  70× | Sniffles2 | 0.7526 | 0.8111 | 0.7807 | **1.0000** | 0.9818 | 0.9908 |
|  | SVIM | **1.0000** | 0.7444 | 0.8535 | **1.0000** | 0.9818 | 0.9908 |
|  | cuteSV | 0.9759 | 0.9 | 0.9364 | **1.0000** | 0.9818 | 0.9908 |
|  | **SVHunter** | **1.0000** | **0.9222** | **0.9595** | **1.0000** | **1.0000** | **1.0000** |
|  | Manta | 0.9545 | 0.2333 | 0.375 | **1.0000** | 0.7091 | 0.8298 |
|  | GRIDSS | **1.0000** | 0.1444 | 0.2524 | 0.9583 | 0.8363 | 0.8932 |
|  | sv-channels | \ | \ | \ | **1.0000** | 0.6909 | 0.8172 |

**Table S5 Benchmark results on simulated illumina data**

|  | SV Type | TP | FN | FP | Precision | Recall | F1 |
| --- | --- | --- | --- | --- | --- | --- | --- |
| Manta | DEL | 608 | 971 | 1317 | 0.3158 | 0.3851 | 0.3470 |
|  | DUP | 231 | 242 | 102 | 0.6937 | 0.4884 | 0.5732 |
|  | INV | 269 | 141 | 214 | 0.5569 | 0.6561 | 0.6025 |
|  | TRS | 298 | 132 | 7612 | 0.0377 | 0.6930 | 0.0715 |
|  | INS | 1 | 1612 | 0 | 1.0000 | 0.0006 | 0.0012 |
|  | ALL | 1407 | 3098 | 9245 | 0.1321 | 0.3123 | 0.1857 |
| GRIDSS | DEL | 759 | 820 | 78 | 0.9068 | 0.4807 | 0.6283 |
|  | DUP | 258 | 215 | 3525 | 0.0682 | 0.5455 | 0.1212 |
|  | INV | 291 | 119 | 141 | 0.6736 | 0.7098 | 0.6912 |
|  | TRS | 312 | 118 | 10021 | 0.0302 | 0.7256 | 0.0580 |
|  | INS | 8 | 1605 | 4 | 0.6667 | 0.0050 | 0.0098 |
|  | ALL | 1628 | 2877 | 13769 | 0.1057 | 0.3614 | 0.1636 |

**Table S6** **Benchmark results on simulated ONT data**

| Tool | SV Type | TP | FN | FP | Precision | Recall | F1 | Weighted F1 |
| --- | --- | --- | --- | --- | --- | --- | --- | --- |
| Sniffles2 | DEL | 1534 | 45 | 44 | 0.9721 | 0.9715 | 0.9718 | 0.3309 |
|  | DUP | 399 | 74 | 0 | **1.0000** | 0.8436 | 0.9151 | 0.0811 |
|  | INV | 6 | 404 | 12 | 0.3333 | 0.0146 | 0.0280 | 0 |
|  | TRS | 326 | 104 | 1098 | 0.2289 | **0.7581** | 0.3517 | 0.0254 |
|  | INS | 1578 | 35 | 498 | 0.7601 | 0.9783 | 0.8555 | 0.2997 |
|  | ALL | 3843 | 662 | 1652 | 0.6994 | 0.8531 | 0.7686 | 0.7371 |
| SVIM | DEL | 1550 | 29 | 25 | 0.9841 | 0.9816 | **0.9829** | 0.3382 |
|  | DUP | 444 | 29 | 0 | **1.0000** | 0.9387 | 0.9684 | 0.0954 |
|  | INV | 404 | 6 | 20 | 0.9528 | 0.9854 | 0.9688 | 0.0869 |
|  | TRS | 196 | 234 | 228 | 0.4623 | 0.4558 | 0.4590 | 0.0200 |
|  | INS | 1567 | 46 | 49 | **0.9697** | 0.9715 | 0.9706 | 0.3376 |
|  | ALL | 4161 | 344 | 322 | 0.9282 | 0.9236 | 0.9259 | 0.8781 |
| cuteSV | DEL | 1553 | 26 | 38 | 0.9761 | **0.9835** | 0.9798 | 0.3378 |
|  | DUP | 450 | 23 | 2 | 0.9956 | 0.9514 | 0.9730 | 0.0972 |
|  | INV | 406 | 4 | 80 | 0.8354 | **0.9902** | 0.9063 | 0.0817 |
|  | TRS | 314 | 116 | 626 | 0.3340 | 0.7302 | 0.4584 | 0.0320 |
|  | INS | 1582 | 31 | 60 | 0.9635 | **0.9808** | **0.9720** | 0.3413 |
|  | ALL | 4305 | 200 | 806 | 0.8423 | **0.9556** | 0.8954 | 0.8899 |
| SVHunter | DEL | 1534 | 45 | 9 | 0.9942 | 0.9715 | 0.9827 | 0.3405 |
|  | DUP | 454 | 19 | 2 | 0.9956 | **0.9598** | **0.9774** | 0.1008 |
|  | INV | 405 | 5 | 14 | 0.9666 | 0.9878 | **0.9771** | 0.0899 |
|  | TRS | 289 | 141 | 9 | **0.9698** | 0.6721 | **0.794** | 0.0642 |
|  | INS | 1561 | 52 | 56 | 0.9654 | 0.9678 | 0.9666 | 0.3465 |
|  | ALL | 4236 | 269 | 86 | **0.9801** | 0.9403 | **0.9598** | 0.9418 |

**Table S7 Benchmark results on simulated CLR data**

| Tool | SV Type | TP | FN | FP | Precision | Recall | F1 |
| --- | --- | --- | --- | --- | --- | --- | --- |
| Sniffles2 | DEL | 1528 | 51 | 93 | 0.9426 | **0.9677** | 0.9550 |
|  | DUP | 357 | 116 | 0 | 1.0000 | 0.7548 | 0.8602 |
|  | INV | 9 | 401 | 8 | 0.5294 | 0.0220 | 0.0422 |
|  | TRS | 288 | 142 | 926 | 0.2372 | **0.6698** | 0.3504 |
|  | INS | 1570 | 43 | 689 | 0.6950 | **0.9733** | 0.8110 |
|  | ALL | 3752 | 753 | 1716 | 0.6862 | 0.8329 | 0.7524 |
| SVIM | DEL | 1500 | 79 | 48 | 0.9690 | 0.9500 | 0.9594 |
|  | DUP | 433 | 40 | 0 | **1.0000** | 0.9154 | 0.9558 |
|  | INV | 378 | 32 | 0 | **1.0000** | 0.9220 | 0.9594 |
|  | TRS | 126 | 304 | 144 | 0.4667 | 0.2930 | 0.3600 |
|  | INS | 1502 | 111 | 66 | **0.9579** | 0.9312 | 0.9444 |
|  | ALL | 3939 | 566 | 258 | 0.9385 | 0.8744 | 0.9053 |
| cuteSV | DEL | 1528 | 51 | 160 | 0.9052 | **0.9677** | 0.9354 |
|  | DUP | 448 | 25 | 1 | 0.9978 | 0.9471 | **0.9718** |
|  | INV | 400 | 10 | 53 | 0.8830 | **0.9756** | 0.9270 |
|  | TRS | 250 | 180 | 354 | 0.4139 | 0.5814 | 0.4836 |
|  | INS | 1553 | 60 | 117 | 0.9299 | 0.9628 | **0.9461** |
|  | ALL | 4179 | 326 | 685 | 0.8592 | **0.9276** | 0.8921 |
| SVHunter | DEL | 1524 | 55 | 30 | **0.9807** | 0.9652 | **0.9729** |
|  | DUP | 444 | 29 | 3 | 0.9933 | 0.9387 | 0.9652 |
|  | INV | 399 | 11 | 6 | 0.9852 | 0.9732 | **0.9791** |
|  | TRS | 233 | 197 | 27 | **0.8962** | 0.5419 | **0.6754** |
|  | INS | 1552 | 61 | 132 | 0.9216 | 0.9622 | 0.9415 |
|  | ALL | 4152 | 353 | 198 | **0.9545** | 0.9216 | **0.9378** |

**Table S8 Benchmark results on simulated CLR data with the same SV type count**

| Tool | SV Type | Precision | Recall | F1 |
| --- | --- | --- | --- | --- |
| Sniffles2 | DEL | 0.8292 | **0.9834** | 0.8997 |
|  | DUP | **1.0000** | 0.7540 | 0.8597 |
|  | INV | 0.6364 | 0.0196 | 0.0380 |
|  | TRS | 0.6667 | 0.5616 | 0.6097 |
|  | INS | 0.4494 | 0.9857 | 0.6174 |
|  | ALL | 0.6532 | 0.6015 | 0.6263 |
| SVIM | DEL | 0.8959 | 0.9669 | 0.9300 |
|  | DUP | **1.0000** | 0.9142 | 0.9552 |
|  | INV | **0.9985** | 0.9230 | 0.9592 |
|  | TRS | 0.6455 | 0.2889 | 0.3991 |
|  | INS | 0.8863 | 0.9481 | 0.9162 |
|  | ALL | 0.8619 | 0.6444 | 0.7374 |
| cuteSV | DEL | 0.7472 | 0.9742 | 0.8457 |
|  | DUP | 0.9945 | 0.8104 | 0.8930 |
|  | INV | 0.8676 | **0.9636** | 0.9131 |
|  | TRS | 0.6135 | 0.4678 | 0.5309 |
|  | INS | 0.7626 | 0.9714 | 0.8544 |
|  | ALL | 0.7486 | 0.7253 | 0.7368 |
| SVHunter | DEL | **0.9401** | **0.9834** | **0.9613** |
|  | DUP | 0.9927 | **0.9233** | **0.9567** |
|  | INV | 0.9754 | 0.9440 | **0.9594** |
|  | TRS | 0.9183 | 0.2588 | 0.4038 |
|  | INS | 0.7090 | 0.9678 | 0.8185 |
|  | ALL | **0.8922** | 0.6402 | **0.7455** |

**Table S9 Benchmark results on HG002 for SV callsets of dfifferent lengths**

| **Length** | **Tool** | **INS** | | | **DEL** | | |
| --- | --- | --- | --- | --- | --- | --- | --- |
|  |  | **Precision** | **Recall** | **F1** | **Precision** | **Recall** | **F1** |
| 50-200 | Sniffles2 | 0.8697 | 0.9639 | 0.9143 | 0.9596 | 0.9248 | 0.9419 |
|  | SVIM | 0.9123 | 0.9515 | 0.9315 | 0.9566 | 0.9157 | 0.9357 |
|  | cuteSV | 0.91 | 0.9357 | 0.9226 | 0.963 | 0.8781 | 0.9186 |
|  | SVHunter | 0.9057 | 0.965 | **0.9344** | 0.9618 | 0.9481 | 0.9549 |
| 200-500 | Sniffles2 | 0.9424 | 0.9471 | 0.9448 | 0.9779 | 0.9876 | 0.9828 |
|  | SVIM | 0.9671 | 0.8744 | 0.9184 | 0.9777 | 0.9777 | 0.9777 |
|  | cuteSV | 0.9626 | 0.9355 | 0.9489 | 0.973 | 0.9802 | 0.9766 |
|  | SVHunter | 0.9529 | 0.9372 | **0.945** | 0.9732 | 0.9876 | 0.9803 |
| 500-1000 | Sniffles2 | 0.8719 | 0.8045 | 0.8369 | 0.9848 | 0.9559 | 0.9701 |
|  | SVIM | 0.929 | 0.7136 | 0.8072 | 0.9265 | 0.9265 | 0.9265 |
|  | cuteSV | 0.9124 | 0.8045 | **0.8551** | 0.9701 | 0.9559 | 0.963 |
|  | SVHunter | 0.9247 | 0.7818 | 0.8473 | 0.971 | 0.9853 | **0.9781** |
| 1000-2000 | Sniffles2 | 0.8539 | 0.5278 | 0.6527 | 1.0000 | 0.9714 | 0.9855 |
|  | SVIM | 0.8846 | 0.3194 | 0.4694 | 1.0000 | 0.9571 | 0.9781 |
|  | cuteSV | 0.887 | 0.7083 | 0.7876 | 1.0000 | 0.9571 | 0.9781 |
|  | SVHunter | 0.8983 | 0.7361 | **0.8092** | 1.0000 | 0.9714 | **0.9855** |
| 2000- | Sniffles2 | 0.0964 | 0.3846 | 0.1542 | 0.9278 | 0.9184 | 0.9231 |
|  | SVIM | 0.875 | 0.0414 | 0.0791 | 0.967 | 0.898 | 0.9312 |
|  | cuteSV | 0.9205 | 0.485 | 0.6353 | 1.0000 | 0.9388 | **0.9684** |
|  | SVHunter | 0.954 | 0.4911 | **0.6484** | 1.0000 | 0.9184 | 0.9575 |

**Table S10 Benchmark results on CHM13 CLR data**

| **Tool** | **Precision** | **Recall** | **F1** |
| --- | --- | --- | --- |
| SVIM | **0.8033** | 0.5977 | 0.6854 |
| Sniffles2 | 0.4274 | **0.8246** | 0.5377 |
| cuteSV | 0.7947 | 0.6305 | 0.7032 |
| SVHunter | 0.7767 | 6662 | **0.7172** |

**Table S11 Benchmark results on CHM13 CLR data with different coverage**

| **Dataset** | **Tool** | **Precision** | **Recall** | | **F1** | |
| --- | --- | --- | --- | --- | --- | --- |
| CHM13  36× | Sniffles2 | **0.8033** | | 0.5977 | | 0.6854 |
|  | SVIM | 0.4274 | | **0.8246** | | 0.5377 |
|  | cuteSV | 0.7947 | | 0.6305 | | 0.7032 |
|  | SVHunter | 0.7767 | | 6662 | | **0.7172** |
| CHM13  20× | Sniffles2 | 0.322 | | **0.6724** | | 0.4355 |
|  | SVIM | **0.8285** | | 0.4439 | | 0.5781 |
|  | cuteSV | 0.8207 | | 0.4804 | | 0.606 |
|  | SVHunter | 0.7559 | | 0.5846 | | **0.6593** |
| CHM13  10× | Sniffles2 | 0.3424 | | **0.5132** | | 0.4107 |
|  | SVIM | 0.8015 | | 0.3183 | | 0.4556 |
|  | cuteSV | **0.8049** | | 0.3351 | | 0.4732 |
|  | SVHunter | 0.7049 | | 0.4563 | | **0.554** |

**Table S12 The false discovered SV records on CHM13 data with different coverage**

| Tool | SV Type | SVHunter | SVIM | Sniffles2 | cuteSV |
| --- | --- | --- | --- | --- | --- |
| 36x | DEL | 29 | 39 | 90 | 51 |
|  | DUP | 94 | 476 | 28825 | 3733 |
|  | INV | 0 | 1 | 2 | 1 |
|  | TRS | 64 | 0 | 1 | 10 |
|  | INS | 68 | 0 | 1 | 109 |
|  | ALL | **255** | 516 | 28919 | 3904 |
| 20x | DEL | 130 | 30 | 93 | 23 |
|  | DUP | 231 | 1766 | 41904 | 1097 |
|  | INV | 0 | 1 | 9 | 0 |
|  | TRS | 94 | 0 | 0 | 8 |
|  | INS | 267 | 0 | 1 | 87 |
|  | ALL | **722** | 1797 | 42007 | 1215 |
| 10x | DEL | 114 | 89 | 46 | 26 |
|  | DUP | 450 | 18098 | 19484 | 1192 |
|  | INV | 4 | 0 | 3 | 0 |
|  | TRS | 174 | 0 | 0 | 4 |
|  | INS | 399 | 0 | 0 | 58 |
|  | ALL | **1141** | 18187 | 19533 | 1280 |

**Table S13 Results on mendelian discordance rate for HG002-HG004**

| SV type | SVHunter | | | SVIM | | | Sniffles2 | | | cuteSV2 | | |
| --- | --- | --- | --- | --- | --- | --- | --- | --- | --- | --- | --- | --- |
|  | Total  calls | Not in parents | MDR  (%) | Total calls | Not in parents | MDR  (%) | Total calls | Not in parents | MDR  (%) | Total calls | Not in parents | MDR |
|  |  |  |  |  |  |  |  |  |  |  |  | (%) |
| DEL | 2801 | 163 | 5.82 | 3481 | 238 | 6.84 | 2992 | 124 | **4.14** | 4111 | 284 | 6.91 |
| INS | 4084 | 327 | **8.01** | 4564 | 387 | 8.48 | 4898 | 564 | 11.51 | 5823 | 632 | 10.85 |
| DUP | 625 | 100 | **16** | 6 | 3 | 50 | 156 | 28 | 17.95 | 513 | 97 | 18.91 |
| INV | 20 | 2 | 10 | 35 | 2 | 5.71 | 93 | 22 | 23.66 | 51 | 15 | 29.41 |
| TRA | 38 | 26 | 68.42 | - | - | - | 422 | 183 | 43.36 | 440 | 37 | 8.41 |
| Total | 7568 | 618 | **8.17** | 8086 | 630 | 7.79 | 8561 | 921 | 10.76 | 10938 | 1065 | 9.74 |

**Table S14 Results on mendelian discordance rate for HG005-HG007**

| SV type | SVHunter | | | SVIM | | | Sniffles2 | | | cuteSV2 | | |
| --- | --- | --- | --- | --- | --- | --- | --- | --- | --- | --- | --- | --- |
|  | Total  calls | Not in parents | MDR  (%) | Total calls | Not in parents | MDR  (%) | Total calls | Not in parents | MDR  (%) | Total calls | Not in parents | MDR |
|  |  |  |  |  |  |  |  |  |  |  |  | (%) |
| DEL | 8984 | 678 | 7.55 | 419 | 32 | 7.64 | 10608 | 808 | 7.62 | 15427 | 809 | 5.24 |
| INS | 17468 | 2518 | **14.41** | 800 | 181 | **22.62** | 22125 | 4211 | 19.03 | 34547 | 8428 | 24.4 |
| DUP | 266 | 44 | **16.54** | 0 | 0 | 0 | 29 | 2 | 6.9 | 353 | 39 | 11.05 |
| INV | 69 | 16 | **23.19** | 1 | 0 | 0 | 101 | 8 | 7.92 | 227 | 13 | 5.73 |
| TRA | 498 | 310 | 62.25 | 0 | 0 | 0 | 1134 | 539 | 47.53 | 980 | 52 | 5.31 |
| Total | 27285 | 3566 | **13.07** | 1220 | 213 | **17.46** | 33997 | 5568 | 16.38 | 51534 | 9341 | **18.13** |

**Table S15 Runtime and memory usage in 28× HG002 HiFi dataset**

| **Tools** | **Threads** | **Elapsed runtime (min)** | **Max memory consumption (KB)** |
| --- | --- | --- | --- |
| SVIM | 1 | 15.38 | 727324 |
| Sniffles2 | 16 | **1.19** | **282380** |
| cuteSV | 16 | 1.39 | [1888100](file:///C:\\Users\\Administrator\\Desktop\\新建%20Microsoft%20Excel%20工作表%20(2).xlsx#RANGE!A8) |
| SVHunter | 16 | 3.53 | 7766900 |

Runtime and memory footprint were assessed by using the "/usr/bin/time -v" command of the Linux Operating System. SVIM does not support multithreading. SVHunter operates in two steps: data generation and variant detection. The recorded times in this study exclude the time spent on data generation.

**Table S16 Benchmark results on A.thaliana using simulated dataset**

| Tool | TP | FN | FP | Precision | Recall | F1 |
| --- | --- | --- | --- | --- | --- | --- |
| cuteSV | 1259 | 491 | 221 | 0.8507 | 0.7194 | 0.7796 |
| Sniffles2 | 1014 | 736 | 330 | 0.7545 | 0.5794 | 0.6555 |
| SVIM | 1260 | 490 | 181 | 0.8744 | **0.72** | 0.7897 |
| SVHunter | 1247 | 503 | 129 | **0.9062** | 0.7126 | **0.7978** |

We evaluated the performance of SVHunter alongside three other tools (cuteSV, Sniffles2, and SVIM) using Arabidopsis thaliana as the reference genome. Due to the lack of high-confidence benchmark datasets or gold standards for most non-human species, including A. thaliana, we employed PBSIM to simulate structural variants (SVs), ensuring a controlled and consistent evaluation. This simulation approach provided a reliable ground truth for assessing precision, recall, and F1-score across different tools.

It is important to clarify that SVHunter was not trained on A. thaliana data prior to conducting the predictions. Instead, the model was trained exclusively on human genomic data, learning generalizable SV features that were then directly applied to A. thaliana without additional retraining. This approach ensures that SVHunter remains independent of species-specific biases, allowing for an objective evaluation of its ability to generalize across different genomes. Based on existing studies in the field, we employed new simulation parameters to better reflect biologically relevant SV distributions. Specifically, we ensured that insertions and deletions together accounted for approximately 80% of the total structural variants, as these are typically the most prevalent SV types. Given the relatively small genome size of A. thaliana, we also adjusted the total number of simulated variants accordingly. The final dataset included 1,000 deletions, 1,000 insertions, 150 inversions, 150 duplications, and 150 translocations, with all structural variants restricted to a maximum length of 1,000 base pairs. The PBSIM commands used for benchmarking are provided in the supplementary materials (Section: Commands used for benchmark) to ensure reproducibility.

The evaluation results indicate that SVHunter achieves the highest F1-score (0.7978) among all tested tools, demonstrating its effectiveness in detecting structural variants in simulated A. thaliana datasets. This performance is driven by its high precision (0.9062) while maintaining a competitive recall (0.7126). Compared to SVIM, which achieved the second-highest F1-score (0.7897, with a precision of 0.8744 and a recall of 0.72), SVHunter exhibited a slightly lower recall but a higher precision, suggesting a more conservative yet accurate variant calling strategy. cuteSV also performed well, attaining an F1-score of 0.7796, but with a lower recall (0.7194) and precision (0.8507) compared to SVHunter. Sniffles2, while still identifying a substantial number of variants, showed the lowest recall (0.5794) and the lowest F1-score (0.6555), indicating potential challenges in detecting certain simulated SVs. Overall, these findings highlight the robustness of SVHunter in detecting structural variants in simulated A. thaliana datasets, achieving a well-balanced performance across both precision and recall. Furthermore, we have updated the supplementary materials to provide a more detailed description of our SV simulation strategy and PBSIM parameters, ensuring transparency and reproducibility of our evaluation.

**Table S17 Benchmark results for A.thaliana across five independent simulated datasets**

| Tool |  | TP | FN | FP | Precision | Recall | F1 |
| --- | --- | --- | --- | --- | --- | --- | --- |
| cuteSV | 1 | 1303 | 447 | 223 | 0.8539 | **0.7446** | 0.7955 |
| Sniffles2 |  | 1045 | 705 | 307 | 0.7729 | 0.5971 | 0.6738 |
| SVIM |  | 1296 | 454 | 160 | 0.8901 | 0.7406 | 0.8085 |
| SVHunter |  | 1287 | 463 | 124 | **0.9121** | 0.7354 | **0.8143** |
| cuteSV | 2 | 1278 | 472 | 256 | 0.8331 | 0.7303 | 0.7783 |
| Sniffles2 |  | 1039 | 711 | 328 | 0.7601 | 0.5937 | 0.6667 |
| SVIM |  | 1295 | 455 | 234 | 0.847 | **0.74** | 0.7899 |
| SVHunter |  | 1288 | 462 | 146 | **0.8982** | 0.736 | **0.809** |
| cuteSV | 3 | 1289 | 461 | 270 | 0.8268 | **0.7366** | 0.7791 |
| Sniffles2 |  | 1042 | 708 | 324 | 0.7628 | 0.5954 | 0.6688 |
| SVIM |  | 1274 | 476 | 208 | 0.8596 | 0.728 | 0.7884 |
| SVHunter |  | 1266 | 484 | 161 | **0.8872** | 0.7234 | **0.797** |
| cuteSV | 4 | 1293 | 457 | 254 | 0.8358 | 0.7389 | 0.7843 |
| Sniffles2 |  | 1059 | 691 | 332 | 0.7613 | 0.6051 | 0.6743 |
| SVIM |  | 1301 | 449 | 191 | 0.872 | 0.7434 | 0.8026 |
| SVHunter |  | 1308 | 442 | 159 | **0.8916** | **0.7474** | **0.8132** |
| cuteSV | 5 | 1258 | 492 | 243 | 0.8381 | 0.7189 | 0.7739 |
| Sniffles2 |  | 998 | 752 | 310 | 0.763 | 0.5703 | 0.6527 |
| SVIM |  | 1268 | 482 | 195 | 0.8667 | **0.7246** | 0.7893 |
| SVHunter |  | 1248 | 502 | 163 | **0.8845** | 0.7131 | **0.7896** |

To further demonstrate the robustness and stability of SVHunter, we generated five independent simulated datasets using the same parameter settings as in our original study. We then re-evaluated the performance of SVHunter, cuteSV, Sniffles2, and SVIM across these datasets. The table presents the F1-score, recall, and precision for each method in each simulation, showing that SVHunter consistently achieves the highest F1-score across all repetitions. These results reinforce its strong predictive performance and reliability in detecting structural variants in A. thaliana.

**Table S18 Benchmark results on A.thaliana using real dataset**

| Tool | TP | FP | FN | Precision | Recall | F1 |
| --- | --- | --- | --- | --- | --- | --- |
| cuteSV | 2724 | 7894 | 625 | 0.2565 | 0.8134 | 0.3901 |
| Sniffles2 | 2924 | 8961 | 425 | 0.2460 | **0.8731** | 0.3839 |
| SVIM | 2660 | 7887 | 689 | 0.2522 | 0.7943 | 0.3828 |
| SVHunter | 2143 | 4302 | 1206 | **0.3325** | 0.6399 | **0.4376** |

Based on a recent study [1], we conducted a benchmark analysis to further validate SVHunter’s performance on real sequencing data. This study provides high-quality chromosome-level genome assemblies for 32 A. thaliana ecotypes, offering a valuable resource for evaluating structural variant (SV) detection tools beyond simulated datasets.

Since the dataset was primarily designed for graph-based pan-genome analysis—focusing on population-level genomic diversity rather than single-sample SV detection—we adapted our benchmarking strategy to better assess the performance of SV detection tools at the individual genome level. First, we selected one of the assembled genomes(meh_0) and aligned it to the reference using minimap2. SVs were then identified using SyRI [2], a widely used tool that supports the detection of various types of genomic variations, including small SNPs and complex variants. We subsequently filtered the resulting VCF file to retain five major types of structural variants for evaluation. It is important to note that although SyRI is a widely adopted tool in the field, the benchmark set it generates may contain certain biases due to limitations inherent to its algorithm. Moreover, the variant calls have not been independently verified through biological experiments, and thus may not fully represent the ground truth. Nevertheless, this benchmark provides a relatively reliable basis for comparing the performance of different SV detection methods. SVHunter achieved the highest F1 score among all evaluated tools, outperforming the second-best method, Sniffles2, by 4.76%. This demonstrates SVHunter’s strong performance on real data.

[1] Kang M, Wu H, Liu H, et al. The pan-genome and local adaptation of Arabidopsis thaliana[J]. Nature Communications, 2023, 14(1): 6259.

[2] Goel M, Sun H, Jiao W B, et al. SyRI: finding genomic rearrangements and local sequence differences from whole-genome assemblies[J]. Genome biology, 2019, 20: 1-13.

**Table S19 Data availability**

| **Data** | **Link** |
| --- | --- |
| The tier1 benchmark SV call set and high-confidence region of HG002 | [https://ftp.ncbi.nih.gov/giab/ftp/data/AshkenazimTrio/analysis/NIST_SVs_Integration_v0.6](https://ftp.ncbi.nih.gov/giab/ftp/data/AshkenazimTrio/analysis/NIST_SVs_Integration_v0.6/) |
| The Ashkenazim trio (including HG002, HG003, and HG004) sequencing reads and bam data | <https://ftp.ncbi.nih.gov/giab/ftp/data/AshkenazimTrio/> |
| The Chinese Trio (including HG005, HG006, and HG007) sequencing reads and bam data | <https://ftp.ncbi.nih.gov/giab/ftp/data/ChineseTrio/> |
| The CLR reads and bam data of CHM13 | <https://github.com/marbl/CHM13> |
| The assembly-based SV call sets of CHM13 | <https://github.com/ldenti/SVDSS-experiments> |
| The A. thaliana reference genome | <https://www.ncbi.nlm.nih.gov/datasets/genome/GCF_000001735.4/> |

**Supplementary Notes**

In this study, we implemented SVHunter using Python as the primary programming language. The feature extraction process was performed with the help of the pysam library, which was used to process BAM files and extract relevant information such as read alignments, mapping quality, and other key features required for structural variant (SV) detection. For the deep learning model, we utilized TensorFlow, a widely used machine learning framework, to design, train, and optimize the architecture. TensorFlow provided the flexibility necessary to build a robust model capable of handling the diverse feature sets extracted from sequencing data. Additionally, standard Python libraries such as NumPy and pandas were employed for data manipulation and preprocessing.

By leveraging these tools, SVHunter was able to efficiently process large-scale sequencing data and achieve accurate SV detection across various datasets. Detailed descriptions of the feature extraction process and model train are provided in the following sections.

1. Feature Extraction

Coverage: indicates the number of reads covering the site;

Deletion CIGAR and Insertion CIGAR: represent the occurrence of deletion ('D') and insertion ('I') operations at the site in the CIGAR field, respectively;

Split read Soft-Match: indicates the presence of a left breakpoint (soft clip, match) at the site;

Split read Match-soft: indicates the presence of a right breakpoint at the site;

Split read Deletion, Insertion, Inversion, Duplication, Translocation: these features represent potential variant types inferred through multiple alignment information.

1. Model train

The model training for SVHunter is based on multiple types of sequencing data from the HG002 sample, including PacBio CLR, CCS and ONT, as well as simulated data based on PacBio CLR and ONT. To enhance the model’s robustness across different coverage depths, we randomly downsampled these data to generate datasets with a coverage depth of 5x for training. During the training process, data from chromosomes 1 to 10 were selected for model training, chromosome 11 was used for validation, and chromosomes 12 to Y were used for testing. The downsampling process ensures that the model maintains high detection performance and accuracy under varying levels of coverage.

In the labeling stage, we used VCF files provided by the Genome in a Bottle Consortium (HG002_SVs_Tier1_v0.6.vcf) as the standard. If any region in the data matrix overlaps with a VCF site from the reference set, that region is labeled as 1, indicating a potential variant; all other non-overlapping regions are labeled as 0. This allows the model to learn from regions with significant variation and improves its ability to detect structural variants. This labeling approach enables SVHunter to more accurately identify variant region boundaries, thereby enhancing the precision of variant detection.

1. Evaluation of SV calling

SVHunter was compared with three advanced SV detection tools: Sniffles2, cuteSV, and SVIM, and the results were validated using Truvari to obtain evaluation metrics such as recall, precision, and F1 score. For SVIM and cuteSV, we adjusted the supporting read parameters according to different coverage levels. For CLR and ONT data, the supporting reads were set to 10 for coverage greater than 40X; for data with 35×, 20×, 10×, and 5x coverage, the supporting reads were set to 5, 4, 3, and 2, respectively. For CCS data, the supporting reads were set to 3, 2, and 1 for 28×, 10×, and 5× coverage, respectively. Sniffles2 and SVHunter support automatic selection of supporting reads, so manual parameter adjustment was not necessary.

To evaluate the results on simulated samples, we used SURVIVOR (v1.0.3) with the eval command, setting the maximum offset error to 500 bp. Precision, recall, and F1 score were calculated based on the true positive (TP), false negative (FN), and false positive (FP) counts reported by SURVIVOR. For the evaluation of the HG002 and CHM13 real samples, we utilized Truvari (v1.3.4). The HG002 structural variantbenchmark set and high-confidence regions were sourced from Genome in a Bottle (GIAB), while the CHM13 SV benchmark set was obtained from a de novo assembly-based SV calling set generated using Dipcall, as described in the recent SVDSS study.

We used the SV call sets from both the Ashkenazi Trio and the Chinese Trio to evaluate the Mendelian Discordance Rate (MDR). For the Ashkenazi Trio, parental sample data for HG003 and HG004 were obtained from the alignment files of 30x PacBio CLR sequencing released by GIAB. For the offspring sample HG002, we selected the alignment file from 35x PacBio CLR sequencing, which has a sequencing coverage comparable to that of the parental samples.

Additionally, we included data from the Chinese Trio (HG005, HG006, and HG007), with an average sequencing coverage of 30x. MDR was calculated as the percentage of SVs in the HG002 and HG007 offspring samples that could not be detected in their respective parental samples (HG003/HG004 and HG005/HG006). The SV discordant was calculated according to equation.

$$MDR=\frac{\sum SV_{discordant}}{\sum SV_{offspring}}$$

Where Σ SV_discordant_ represents the total number of SVs in the offspring sample that are not supported by the parental samples, and Σ SV_offspring_ is the total number of SVs detected in the offspring sample. SVs in the offspring sample were compared to the parental SV call sets based on their genomic coordinates, type, and size. Variants that could not be matched with parental SVs were classified as discordant. A lower MDR value indicates better Mendelian consistency, suggesting that most SVs in the offspring sample are supported by the parental samples. Conversely, a higher MDR value may indicate false positive calls, insufficient sequencing coverage, or inconsistencies in SV detection across samples.

1. Additional considerations in CHM13 benchmarking

**4.1 The relative merits and drawbacks of assembly versus non-assembly-based method**

While de novo assembly-based methods, such as Dipcall, are widely regarded as highly accurate and trustworthy for structural variant (SV) detection, non-assembly-based methods like SVHunter offer several complementary advantages. These advantages include:

(1) Efficiency and Scalability:

Non-assembly-based methods require significantly less computational time and resources compared to assembly-based methods [1]. De novo assembly is computationally intensive, particularly for large and complex genomes, and may not be practical for routine analysis or large-scale studies involving hundreds or thousands of samples.

(2) Applicability to Low-Coverage Data:

Assembly-based methods generally require high-coverage sequencing data to produce accurate results. In contrast, non-assembly-based methods like SVHunter are designed to perform well even under lower coverage conditions (e.g., 5×), making them more suitable for applications where high-coverage data may not be feasible or available [2].

(3) Broader Accessibility:

Non-assembly-based methods are more accessible to researchers with limited computational resources or expertise in genome assembly [3,4]. This makes them a practical choice for broader adoption in diverse research and clinical settings.

In summary, while assembly-based methods like Dipcall provide a valuable benchmark for SV detection, non-assembly-based methods such as SVHunter offer distinct advantages in terms of efficiency, scalability, and applicability to diverse datasets. These methods can be particularly advantageous in real-world scenarios where computational resources or high-coverage data are limited, and they serve as complementary approaches to enhance the overall SV detection landscape.

**4.2 Justification for using CHM13 as a gold-standard reference.**

This paper chose CHM13 as a benchmark for this experiment because it represents one of the most accurate and complete reference genomes currently available, especially in resolving highly repetitive regions. Compared to other references (e.g., GRCh38), CHM13 provides a more reliable baseline for assessing the false-positive resistance of SV detection tools [4].

Regarding the potential errors in the assembly process, we note that CHM13 was constructed with state-of-the-art telomere-to-telomere (T2T) assembly technologies, which have been shown to produce extremely accurate results with a very low rate of errors. For example, recent studies ([5-6]) have demonstrated that T2T assembly can resolve nearly all repetitive regions and significantly outperform traditional alignment-based methods. While minor errors in the assembly process cannot be entirely excluded, their frequency and impact are minimal compared to the overall accuracy of the CHM13 assembly.

As for somatic variants, CHM13 is derived from a hydatidiform mole, which is effectively a haploid genome. This means that the likelihood of somatic variants arising from cellular heterogeneity is extremely low. While it is true that somatic variants could theoretically exist in a small fraction of cells, our goal in this experiment was not to study somatic variants but rather to evaluate the ability of SVHunter to filter out false-positive calls in a high-quality, near-error-free genome. Given the nature of the CHM13 sample, we believe it provides a robust benchmark for this purpose.

It is important to note that we do not claim CHM13 is entirely error-free. Instead, we used it to provide a controlled environment for testing the false-positive resistance of SV detection tools. The results demonstrated that SVHunter detected significantly fewer false-positive SVs compared to other tools (Figure 4B). This aligns with the objective of our study, which is to evaluate and optimize SVHunter's performance across different scenarios.

**4.3 Why detecting fewer SVs does not indicate poor performance**

A common concern in structural variant (SV) detection is the possibility of a "bad oracle"—a method that appears to perform well in false-positive resistance evaluations simply by calling very few or no SVs, even if it fails to detect true variants. However, SVHunter's performance is not based on minimizing SV calls but rather on achieving a balance between precision and recall, as demonstrated through multiple experiments on both real and simulated datasets.

(1) Evaluation beyond false positives

To assess a tool’s ability to filter out false-positive SVs in a high-quality genome, we conducted an experiment aligning the CHM13 sample to the CHM13 reference genome. Since CHM13 should ideally contain no SVs relative to itself, recall was not a relevant metric in this specific test. This experiment was designed to evaluate false-positive resistance rather than overall SV detection capability.

(2) Validation across multiple datasets

To ensure that SVHunter is not simply under-calling variants, we evaluated its performance on multiple datasets, including both real and simulated data, where both precision and recall were considered. The consistently high F1 scores across these datasets demonstrate that SVHunter maintains a balance between precision and recall rather than just minimizing SV calls.

(3) Comparison with prior studies

Similar approaches have been used in previous studies to evaluate false-positive resistance. For example, SVDSS employed this strategy to assess SV detection tools, while the somatic SV detection tool Savana utilized a comparable method by splitting a tumor sample into two equal sequencing-depth parts to evaluate performance. These precedents highlight the value of controlled experimental designs in assessing the robustness and specificity of SV detection tools.

**4.4 Limitations of overlap analysis in False-Positive resistance evaluation**

In this study, we considered whether the overlap between structural variants (SVs) detected by different tools could indicate potential true positives or assembly errors. However, such an analysis is not applicable in the context of our second experiment.

The purpose of this experiment was to evaluate the false-positive resistance of SV detection tools using the CHM13 genome, a telomere-to-telomere (T2T) assembly with extremely high accuracy. CHM13 is one of the most complete and high-quality human genome assemblies available [4], serving as a near-error-free reference. Thus, any SVs detected when aligning CHM13 sequencing data to the CHM13 reference genome are expected to predominantly represent systematic false positives due to tool limitations rather than true structural variants. While CHM13 is highly accurate, small assembly errors or unresolved regions cannot be entirely ruled out.

It is important to note that this approach is not unique to our study; similar methodologies have been successfully applied in previous research to evaluate the performance of SV detection tools. For example, SVDSS employed this strategy by aligning sequencing data to a high-quality reference genome and interpreting detected SVs as false positives [7]. Likewise, the somatic structural variant detection tool Savana used a comparable approach by splitting a tumor sample into two equal parts with the same sequencing depth to benchmark tool performance [8]. These studies demonstrate that our methodology is well-established for assessing false-positive resistance in SV detection tools.

Since both the query data and the reference genome originate from the same CHM13 assembly in this experiment, any detected SVs cannot represent real genomic variations [9]. Instead, they likely reflect tool-specific biases, alignment artifacts, or systematic errors in unresolved or repetitive regions. Previous studies have shown that such regions—centromeres, telomeres, and segmental duplications—are common sources of false-positive SVs and areas where minor assembly errors are most likely to occur [10,11]. Consequently, overlap analysis in this experiment would provide limited value, as it would primarily highlight systematic false positives rather than distinguish between them and potential assembly errors.

In summary, while overlap analysis between SV detection tools can be informative in other experimental contexts, it is not suitable for this study. The detected SVs predominantly arise due to tool-specific biases and unresolved regions in the CHM13 assembly. Instead, our approach—successfully applied in prior studies—provides a robust evaluation of tool-specific false-positive resistance and highlights the challenges of SV detection in repetitive genomic regions.

[1] Mills R E, Walter K, Stewart C, et al. Mapping copy number variation by population-scale genome sequencing[J]. Nature, 2011, 470(7332): 59-65.

[2] Sedlazeck F J, Rescheneder P, Smolka M, et al. Accurate detection of complex structural variations using single-molecule sequencing[J]. Nature methods, 2018, 15(6): 461-468.

[3] Liu Y H, Luo C, Golding S G, et al. Tradeoffs in alignment and assembly-based methods for structural variant detection with long-read sequencing data[J]. Nature Communications, 2024, 15(1): 2447.

[4] Mahmoud M, Gobet N, Cruz-Dávalos D I, et al. Structural variant calling: the long and the short of it[J]. Genome biology, 2019, 20: 1-14.

[4] Nurk S, Koren S, Rhie A, et al. The complete sequence of a human genome[J]. Science, 2022, 376(6588): 44-53.

[5] Miga K H, Koren S, Rhie A, et al. Telomere-to-telomere assembly of a complete human X chromosome[J]. Nature, 2020, 585(7823): 79-84.

[6] Li H, Durbin R. Genome assembly in the telomere-to-telomere era[J]. Nature Reviews Genetics, 2024: 1-13.

[7] Denti L, Khorsand P, Bonizzoni P, et al. SVDSS: structural variation discovery in hard-to-call genomic regions using sample-specific strings from accurate long reads[J]. Nature Methods, 2023, 20(4): 550-558.

[8] Elrick H, Sauer C M, Espejo Valle-Inclan J, et al. SAVANA: reliable analysis of somatic structural variants and copy number aberrations in clinical samples using long-read sequencing[J]. bioRxiv, 2024: 2024.07. 25.604944.

[9] Chaisson M J P, Huddleston J, Dennis M Y, et al. Resolving the complexity of the human genome using single-molecule sequencing[J]. Nature, 2015, 517(7536): 608-611.

[10] Ebert P, Audano P A, Zhu Q, et al. Haplotype-resolved diverse human genomes and integrated analysis of structural variation[J]. Science, 2021, 372(6537): eabf7117.

[11] Sedlazeck F J, Lee H, Darby C A, et al. Piercing the dark matter: bioinformatics of long-range sequencing and mapping[J]. Nature Reviews Genetics, 2018, 19(6): 329-346.

1. Commands used for benchmark

**5.1 read alignment**

*> minimap2 -ax map-pb fast.fa *.fastq > sample.sam*

*> samtools sort -m4G -@16 -o sample.bam sample.sam*

*> samtools index sample.bam*

**5.2 SV simulation**

*> SURVIVOR simSV parameter_file*

*> SURVIVOR simSV ref.fa parameter_file 0.1 0 simulated*

*> art_illumina -p -sam -I simulated.fasta -l 150 -f 100 -m 200 -s 10 -o ./artsim/*

*> pbsim --depth 30 --hmm_model P6C4.model simulated.fasta*

*> pbsim --depth 30 --hmm_model R103.model simulated.fasta*

**5.3 SV calling**

For SVIM:

*> svim alignment vcfpath bamfile.bam reference.fa*

For Sniffles2:

*> sniffles --threads 15 --input bamfile.bam -- vcf variant.vcf*

For cuteSV:

*> cuteSV --genotype --threads 20 -s min_supporting_reads bamfile.bam reference.fa ./variant.vcf tmpdir*

For Manta:

*> configManta.py --bam bafile.bam* *--referenceFasta reference.fa --runDir dir*

For Gridss:

*> gridss -r reference.fa* *-j reference.fa -o output.vcf input1.bam input2.bam*

For sv-channels:

*> svchannels extract-signals reference.fasta sample.bam -o signals*

*>* *Rscript svchannels/utils/R/vcf2bedpe.R -i manta.vcf -o manta.bedpe*

*> svchannels generate-channels --reference reference.fasta signals channels manta.bedpe*

*> svchannels score channels model.keras manta.vcf sv-channels.vcf*

**5.4 SV call**

For Truvari:

*> truvari --passonly -p 0 --sizefilt 50 --sizemin 50 --includebed HG002_SVs_Tier1_v0.6.bed -b HG002_SVs_Tier1_v0.6_del_12_Y.vcf.gz -c sample.vcf -f reference.fa -o ./result*

For SURVIVOR:

*> SURVIVOR eval variants.vcf sim.bed 500 eval_res*

**5.5 Pan-Genomic Structural Variation Detection in Arabidopsis thaliana**

*> minimap2 -ax asm5 -t 64 --eqx GCF_output.fna 44.ket_10.fasta > ket_10.sam*

*> samtools sort -m4G -@64 -o ket_10.bam ket_10.sam*

*> syri -c ket_10.sam -r GCF_output.fna -q 44.ket_10.fasta -k -F S*
